# Cotrimoxazole Prophylaxis and Risk of Severe Anemia or Severe Neutropenia in HAART-Exposed, HIV-Uninfected Infants

**DOI:** 10.1371/journal.pone.0074171

**Published:** 2013-09-23

**Authors:** Scott Dryden-Peterson, Oluwemimo Jayeoba, Michael D. Hughes, Haruna Jibril, Kenneth McIntosh, Taolo A. Modise, Aida Asmelash, Kathleen M. Powis, Max Essex, Roger L. Shapiro, Shahin Lockman

**Affiliations:** 1 Division of Infectious Diseases, Brigham and Women’s Hospital, Boston, Massachusetts, United States of America; 2 Botswana Harvard AIDS Institute Partnership, Gaborone, Botswana; 3 Department of Immunology and Infectious Diseases, Harvard School of Public Health, Boston, Massachusetts, United States of America; 4 Department of Biostatistics, Harvard School of Public Health, Boston, Massachusetts, United States of America; 5 Department of Public Health, Ministry of Health, Gaborone, Botswana; 6 Division of Infectious Diseases, Boston Children’s Hospital, Boston, Massachusetts, United States of America; 7 Departments of Internal Medicine and Pediatrics, Massachusetts General Hospital, Boston, Massachusetts, United States of America; 8 Division of Infectious Diseases, Beth Israel Deaconess Medical Center, Boston, Massachusetts, United States of America; University of New South Wales, Australia

## Abstract

**Background:**

Prophylactic cotrimoxazole is recommended for infants born to HIV-infected mothers. However, cotrimoxazole may increase the risk of severe anemia or neutropenia.

**Methods:**

We compared the proportion of HIV-exposed uninfected (HIV-EU) infants experiencing incident severe anemia (and separately, severe neutropenia) between a prospective cohort receiving prophylactic cotrimoxazole from 1 to 6 months vs. infants from two prior trials who did not receive cotrimoxazole. Infants were from rural and urban communities in southern Botswana.

**Results:**

A total of 1705 HIV-EU infants were included. Among these 645 (37.8%) were fed with iron-supplemented formula from birth. Severe anemia developed in 87 (5.1%) infants, and severe neutropenia in 164 (9.6%) infants. In an analysis stratified by infant feeding method, there were no significant differences in the risk of severe anemia by prophylactic cotrimoxazole exposure–risk difference, −0.69% (95% confidence interval [CI] −2.1 to 0.76%). Findings were similar in multivariable analysis, adjusted odds ratio (aOR) 0.35 (95% CI 0.07 to 1.65). There were also no significant differences observed for severe neutropenia by cotrimoxazole exposure, risk difference 2.0% (95% CI −1.3 to 5.2%) and aOR 0.80 (95% CI 0.33 to 1.93).

**Conclusions:**

Severe anemia and severe neutropenia were infrequent among HIV-exposed uninfected infants receiving cotrimoxazole from 1–6 months of age. Concerns regarding hematologic toxicity should not limit the use of prophylactic cotrimoxazole in HIV-exposed uninfected infants.

**ClinicalTrials.gov Registration Numbers:**

NCT01086878 (http://clinicaltrials.gov/show/NCT01086878), NCT00197587 (http://clinicaltrials.gov/show/NCT00197587), and NCT00270296 (http://clinicaltrials.gov/show/NCT00270296).

## Introduction

The World Health Organization (WHO) recommends that all infants born to HIV-infected mothers receive prophylactic cotrimoxazole until they are known to be HIV-uninfected and they are no longer at risk of acquiring HIV via breastfeeding. [1] Due to challenges with timely infant HIV diagnosis, the period of recommended cotrimoxazole use for the majority of the nearly two million HIV-exposed infants born in sub-Saharan Africa annually [2] exceeds 6 months for formula-fed infants and is greater than 12 months for a majority of breastfed infants. However, with maternal highly active antiretroviral therapy (HAART), less than 2% of infants acquire HIV infection. [3]–[5] With improved access to maternal HAART during pregnancy and breastfeeding, [6] the recommendation to provide empiric cotrimoxazole prophylaxis for all HIV-exposed infants has been questioned. [7].

Maternal HAART has been associated with increased risk of infant anemia[8]–[11] [12] and neutropenia. [13], [14] We previously found an association between exposure to maternal HAART and severe infant anemia [12]. Post-natal prophylactic infant zidovudine likely further increases this risk. [15] Cotrimoxazole also can adversely affect hematopoiesis, [16], [17] although virtually no data are available for the effect in infants. [18] The combination of HAART and cotrimoxazole appears to increase risk of hematologic toxicity in sub-Saharan African adults. [19]–[21] Infants may be more vulnerable to this effect, as hemoglobin concentration falls following birth reaching a physiologic nadir between 1 and 2 months of life before recovering by 6 months. [22], [23] Provision of prolonged cotrimoxazole prophylaxis to young infants may potentiate anemia and neutropenia in HAART-exposed infants.

An increased risk of severe hematologic complications could alter the balance of risks and benefits of the WHO strategy of cotrimoxazole for all HIV-exposed infants. Additionally, there is interest in using cotrimoxazole prophylaxis to reduce excess morbidity in HIV-exposed uninfected (HIV-EU) infants. We therefore sought to prospectively compare the proportion of infants with incident severe anemia and incident severe neutropenia from one to six months of age between HIV-exposed infants receiving cotrimoxazole prophylaxis in a new cohort (CTX) and infants not receiving cotrimoxazole prophylaxis in two prior similar cohorts (CTX-unexposed).

## Methods

### Ethics Statement

All participating mothers provided written informed consent for themselves and on behalf of their infants. The studies were reviewed and approved by the Botswana Health Research Development Committee and the Institutional Review Board of the Harvard School of Public Health. The trial clinicaltrials.gov registration numbers are NCT01086878, NCT00197587, and NCT00270296. The protocol for this trial and supporting CONSORT checklist are available as supporting information; see Checklist S1 and Protocol S1.

### Study Design

We performed a prospective, historically-controlled clinical trial of prophylactic cotrimoxazole. We compared the frequency of hematologic toxicity between HIV-EU infants in a new prospective cohort (CTX) that received cotrimoxazole prophylaxis, and HIV-EU infants in two prior studies (Mashi and Mma Bana) that did not receive cotrimoxazole prophylaxis (CTX-unexposed). Infants were followed in the same communities and clinics, and resided in non-malarial regions of Botswana.

**Figure 1:**
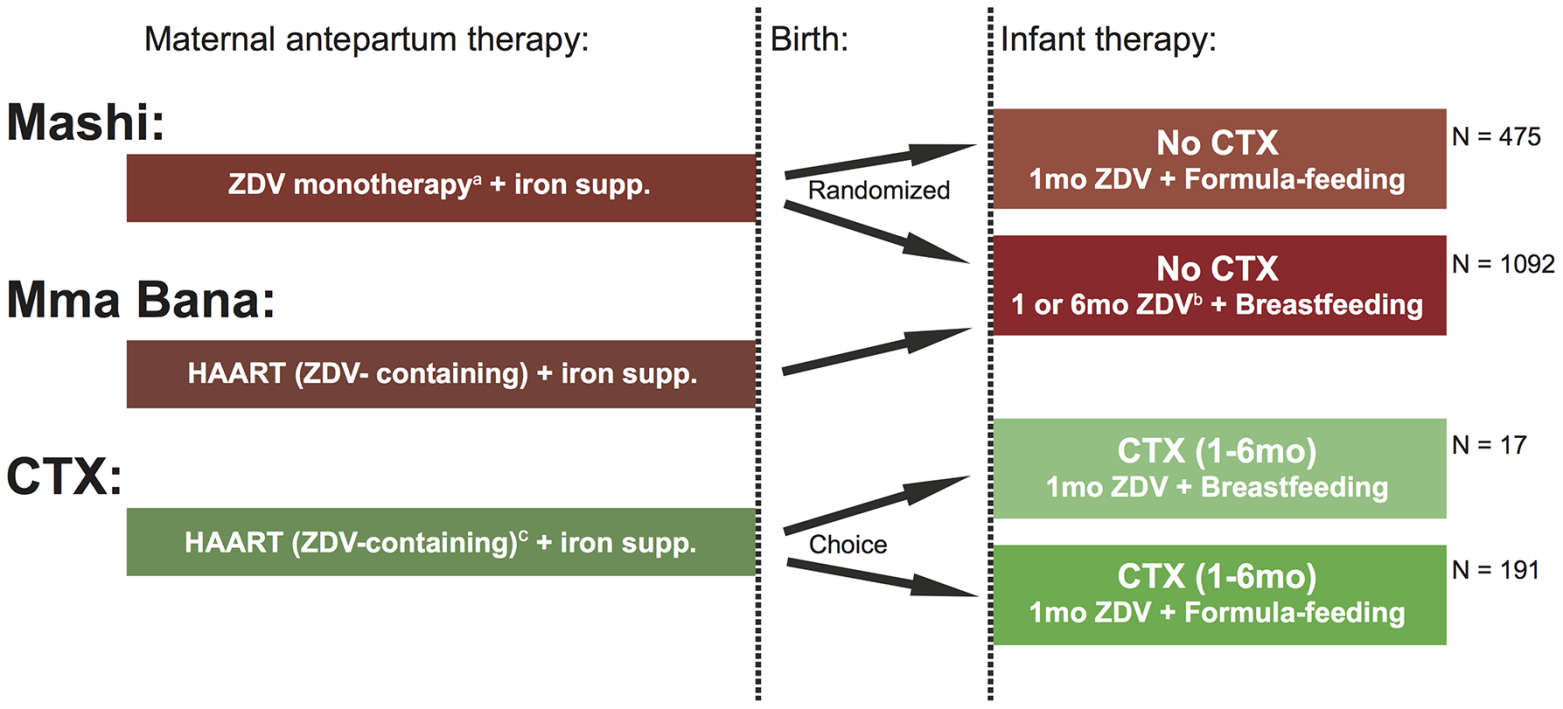
Schematic of infant exposures in study cohorts. ZDV, zidovudine; supp., supplementation; CTX, cotrimoxazole; HAART, highly-active antiretroviral therapy; mo., month. ^a^HAART became available through a national program in October 2002, subsequently women in Mashi trial with CD4≤200 cells/ µL were offered HAART.^ b^Infants in the Mma Bana trial received 1 month of ZDV and breastfed infants in the Mashi trial received 6 months of ZDV.^ c^Nineteen mothers (9.1%) in CTX cohort received non-ZDV-containing HAART.

To emulate current practice where infant HIV diagnosis may be delayed until beyond 6 months, HIV-EU infants in the CTX cohort were assigned to receive cotrimoxazole (Purbac suspension, Aspen Pharmacare Limited, Johannesburg, South Africa) from 1 to 6 months of age, regardless of feeding method. Recommended weight-based dosing of cotrimoxazole was used (<5 kg, 100 mg sulfamethoxazole/20 mg trimethoprim once daily; ≥5 kg, 200 mg sulfamethoxazole/40 mg trimethoprim once daily). [1], [24] The frequencies of anemia and neutropenia in this cohort were compared with frequencies of these outcomes among HIV-EU infants in the CTX-unexposed cohort which did not provide cotrimoxazole prophylaxis to uninfected infants (Figure 1).

### Study Subjects

For the CTX cohort, we approached HIV-infected mothers receiving antenatal HAART who delivered live-born infants between February 2009 and April 2010. According to national guidelines at the time of the study, [24] women with CD4 cell count ≤250 cells/ µL or WHO clinical stage 3 or 4 were eligible for HAART. Women were provided with feeding counseling, and were eligible for the study whether they opted to breastfeed or formula-feed. Infants born to consenting mothers were enrolled in the CTX cohort when they returned to the study clinic at one month of age. The Mashi and Mma Bana trials enrolled pregnant women and their infants. The HIV-uninfected infants from these trials were included in the CTX-unexposed cohort if they attended the scheduled clinic visit at one month of age.

All infants were recommended to receive single-dose nevirapine at birth and 4 weeks of zidovudine prophylaxis. Breastfeeding infants in the Mashi trial were assigned to receive extended zidovudine through 6 months. Breastfeeding mothers in all cohorts were counseled to wean at 6 months. Replacement-fed infants in all cohorts received iron-fortified formula free of charge through the Botswana national program and mother received prenatal iron supplementation. Immediate cord clamping was the standard obstetrical practice during all three studies. Interviews with midwives caring for subjects in all three cohorts indicate that there has not been a change in maternal or infant supplements. Details of maternal and infant interventions of the cohorts are summarized in Table S1.

Adherence to cotrimoxazole was assessed through self-report and pharmacy refill records. Infants were weighed at each visit to confirm appropriate dosage. All women were taught by a nurse at each visit about the appropriate dose to administer.

### Laboratory Measurements

Infants had laboratory measurements at birth and at 1, 3, and 6 months (other than for Mashi formula-fed infants who were seen at birth and at 1, 4, and 7 months). Peripheral blood for a full blood count with differential was taken at each scheduled visit (no measurement at birth in CTX cohort). Full blood counts were performed using the Sysmex XE 2100 automated flow cytometry analyzer (Sysmex Corporation, Kobe, Japan). Infant HIV testing was performed at similar intervals by qualitative polymerase chain reaction (PCR) DNA assay using the Amplicor HIV-1 test (Roche Diagnostic Systems, New Jersey).

### Endpoints and Exposures

The primary endpoint was the first episode of severe (grade 3 or 4) anemia (or first episode of grade 3 or 4 neutropenia in a separate analysis). Grading was according to the Division of AIDS (DAIDS) toxicity tables, 2004 revision, developed from normative ranges from the United States and Europe and expert opinion. [25] To reduce ascertainment bias, only severe anemia or neutropenia detected at scheduled 3 and 6 month visits (4 and 7 month visits in Mashi formula-fed group) were considered as endpoints. Infants born to mothers taking HAART starting at least one day prior to delivery were defined as HAART-exposed and infants that did not initiate breastfeeding prior to hospital discharge were considered as formula-fed. Infants that initiated breastfeeding, but subsequently stopped prior to 6 months of age were considered as breastfed for purposes of analysis.

### Analytic Methods

The study was originally designed to detect a clinically significant increase (10 percent absolute increase) in the proportion of breastfed, HIV-exposed uninfected (HIV-EU) infants with severe anemia between infants receiving versus not receiving cotrimoxazole. However, with increasing governmental support for replacement feeding of HIV-EU infants in Botswana, we were unable to enroll sufficient numbers of HIV-EU breastfed infants for this comparison. Consequently, we opened enrollment in the CTX cohort to both breastfed and formula-fed HIV-EU infants. Among formula-fed infants, the modified design had greater than 90 percent power to detect an absolute 5 percent increase (above the observed 1 percent in the Mashi trial) in the proportion with severe anemia.

Analyses were restricted to HIV-uninfected infants. Stratifying by infant feeding method, we used an exact Cochran-Mantel-Haenszel test to examine for significant differences in cumulative incident cases of severe anemia and severe neutropenia by cotrimoxazole group. The Breslow-Day test was used to guide decision of pooling stratified odds ratios. Multiple logistic regression was used subsequently to adjust for baseline differences between the cotrimoxazole exposure groups. Cotrimoxazole assignment, antenatal maternal HAART, and infant feeding method were retained in the model. Backwards stepwise model selection, retaining all factors significantly associated (*P*<0.05) with the outcome, was used to determine the final multivariable model. Statistical analyses were performed using SAS, version 9.2 (SAS Institute, Cary, NC). All tests were 2-tailed, *P* values of less than 0.05 were considered statistically significant.

## Results

### Study Infants

**Figure 2:**
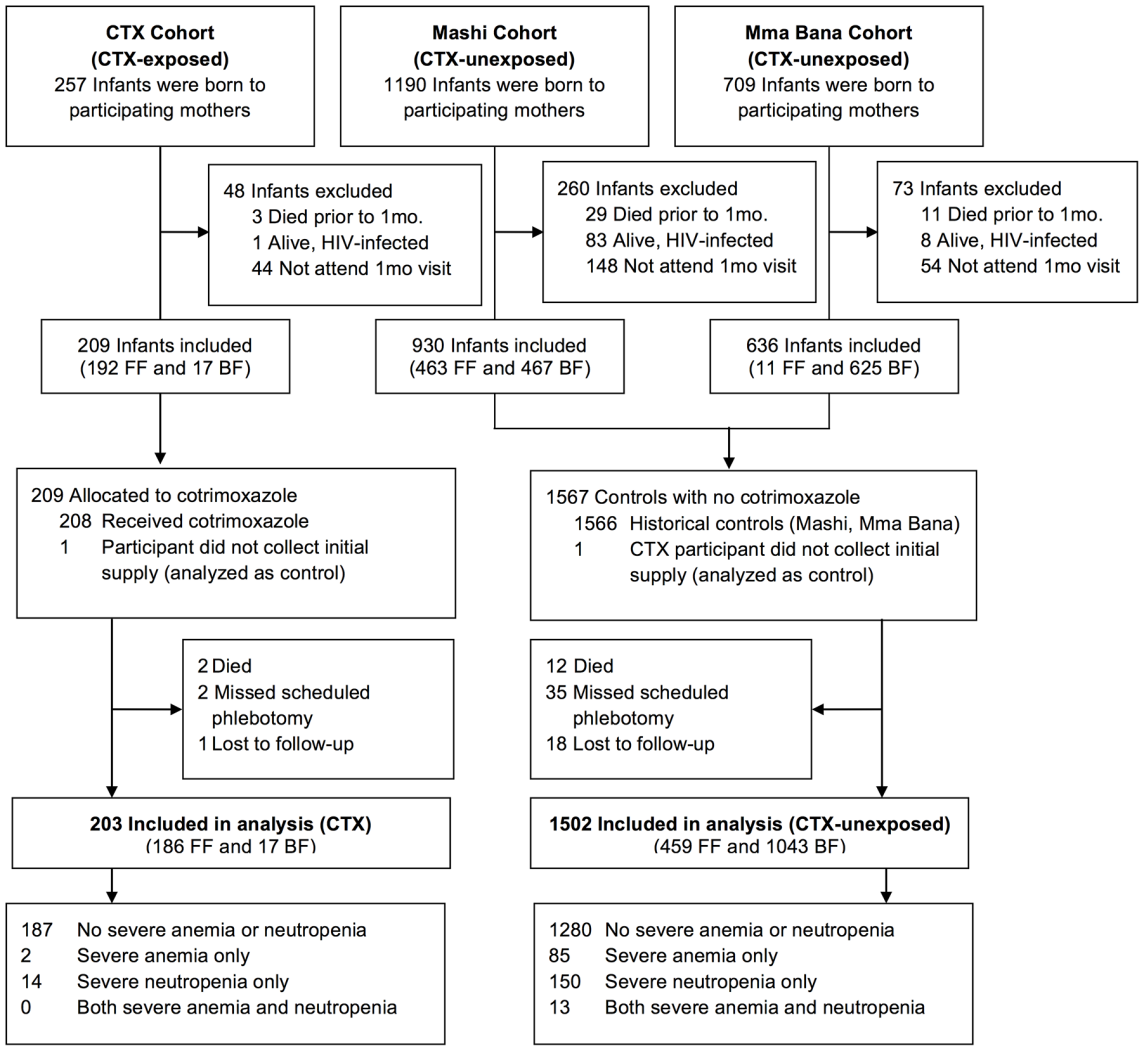
Enrollment and follow-up of study infants. HIV-uninfected infants in the Mashi and Mma Bana trials did not receive cotrimoxazole prophylaxis and serve as a comparison group to the new cohort (CTX) that received cotrimoxazole prophylaxis. CTX, cotrimoxazole; 1 mo., one month; FF, formula-fed; BF, breastfed.

There were 2156 live infants (257 CTX, 1899 CTX-unexposed) including 23 sets of twins, born to 2133 HIV-infected women in the study cohorts. A total of 93 infants had detected HIV infection through 7 months of age and were excluded from analysis. Forty-two infants who died prior to 1 month of age and 246 infants who did not attend the 1 month visit were also excluded. A total of 1775 infants were included in the cohort. Seventy infants did not have available hematology measurements at 3 or 6 months of age, leaving 1705 analyzable infants (203 CTX, 1502 CTX-unexposed). One CTX infant did not initiate cotrimoxazole (Figure 2).

Maternal and infant characteristics are summarized in Table 1. A total of 645 (37.8%) of infants formula-fed from birth, while 1060 (62.2%) breastfed from birth. Mothers of CTX infants had higher incomes, more education, and higher prevalence of antenatal HAART exposure compared with mothers of infants not receiving cotrimoxazole (reflecting differences in study design and eligibility criteria for participants in these studies). Perhaps as a consequence of maternal HAART (which was more frequently used in the CTX cohort than in the historical cohorts), [12], [26], [27] CTX infants were more frequently premature and small for gestational age, and had lower hemoglobin concentrations at 1 month than the infants not receiving cotrimoxazole.

**Table 1:** Cohort characteristics of HIV-exposed uninfected infants alive through 30 days of age.

		Cotrimoxazole Prophylaxis (CTX)	No Cotrimoxazole Prophylaxis (CTX-unexposed)
		N = 208	N = 1567
		n (%)	n (%)
Village residence		96 (46.2)	802 (51.2)
Maternal income			
	None	68 (32.7)	869 (56.1)
	<$100	89 (42.8)	370 (23.9)
	≥$100	51 (24.5)	311 (20.1)
Maternal education			
	Primary or none	52 (25.0)	399 (25.7)
	Secondary	140 (67.3)	1101 (71.0)
	University	16 (7.7)	51 (3.3)
Maternal HAART		208 (100)	696 (44.4)
Maternal age (years), median (IQR)		31.7 (27.6, 35.0)	27.1 (23.4, 31.6)
Maternal CD4 (cells/ µL), median (IQR)		278 (202, 421)	362 (238, 503)
Male infant		105 (50.5)	808 (51.6)
Premature (<37 weeks)		45 (21.6)	202 (13.1)
Small for gestational age (<10 percentile)		47 (22.6)	177 (11.5)
Breastfed at discharge from maternity ward		17 (8.2)	1092 (69.7)
Infant hemoglobin (g/dL) at 1 month, median (IQR)		10.9 (9.9, 11.7)	11.1 (10.1, 12.2)
Infants with available hematology measurements		203 (97.6)	1502 (95.9)

Note: CTX, cotrimoxazole; IQR, interquartile range.

### Completeness of Hematology Data

Hematologic measurements were available at either 3 or 6 months for 96% of infants. Measurements at both of these visits were available for 81%. There were not significant differences in completeness of study measurements by exposure to cotrimoxazole or maternal HAART, feeding method, or study cohort.

### Adherence to Cotrimoxazole

Among infants initiating cotrimoxazole, 94% completed the prophylaxis through 6 months as assessed through pharmacy refills. Reasons for premature discontinuation in the 13 infants were as follows: death (3 cases of gastroenteritis and 1 case of possible sudden infant death syndrome), mild rash (1), mild persistent diarrhea (1), mother opted to stop medication without specifying reason (1), and did not return for medication refill (6).

### Primary Outcomes

#### Severe anemia

Protocol-defined severe anemia (grade 3 or 4) developed in 2 CTX infants (1.0%) and in 85 CTX-unexposed infants (5.7%). Nearly all infants with severe anemia (93%) were breastfed. In an analysis stratified by infant feeding method, there were no significant differences in the risk of severe anemia by prophylactic cotrimoxazole exposure – odds ratio 0.57 (95% confidence interval [CI] 0.12 to 2.77), exact Cochran-Mantel-Haenszel *P* = 0.75). The stratified estimates of the risk difference, −0.69% (95% CI -2.1 to 0.76%), indicate that a clinically significant increase in the risk of severe anemia with cotrimoxazole exposure is unlikely (Table 2).

**Table 2:** Severe infant anemia and severe neutropenia by prophylactic cotrimoxazole exposure.

	Cotrimoxazole Prophylaxis	No Cotrimoxazole Prophylaxis
	(CTX)	(CTX-unexposed)
	N = 203	N = 1502
**Severe Anemia**		
Breast-fed infants	0/17 (0%)	81/1043 (7.6%)
Formula-fed infants	2/186 (1.1%)	4/459 (0.9%)
Stratified pooled estimates^a^		
Odds ratio (95% CI)	0.57 (0.12 to 2.77), P = 0.75^b^	
Risk difference (95% CI)	−0.69% (−2.1% to 0.76%)	
**Severe Neutropenia**		
Breast-fed infants	3/17 (17.7%)	130/1040 (12.5%)
Formula-fed infants	11/186 (5.9%)	20/458 (4.4%)
Stratified pooled estimates^a^		
Odds ratio (95% CI)	1.41 (0.73 to 2.69), P = 0.29^b^	
Risk difference (95% CI)	2.0% (−1.3% to 5.2%)	

Analysis restricted to severe anemia or neutropenia (grade 3 or 4) detected at scheduled measurements at 3 and/or 6 months of age in HIV-exposed uninfected infants in the CTX, Mashi, and Mma Bana cohorts.

Note: 95% CI, 95% confidence interval.

a Mantel-Haenszel methodology.

b Exact Cochran-Mantel-Haenszel.

Findings in multivariable analyses were similar. In a model adjusted for infant feeding method, antenatal maternal HAART, and other significant baseline factors, the estimated adjusted odds ratio (aOR) for severe anemia for CTX versus CTX-unexposed was 0.35 (0.07 to 1.65), P = 0.18. In this model, breastfeeding, antenatal maternal HAART, male sex, and low maternal income were all significantly associated with increased risk of severe anemia (Table 3).

**Table 3:** Factors associated with severe anemia and severe neutropenia among HIV-exposed, uninfected infants.

Variable	Severe Anemia				Severe Neutropenia			
	Univariate		Multivariable		Univariate		Multivariable	
	OR (95% CI)	P-value^a^	aOR (95% CI)	P-value^a^	OR (95% CI)	P-value^a^	aOR (95% CI)	P-value^a^
Infant Cotrimoxazole, 1 to6 months^b^	1.24 (0.23–6.81)	0.81	0.35 (0.07–1.65)	0.18	1.38 (0.65–2.93)	0.41	0.80 (0.33–1.93)	0.62
Maternal Antenatal HAART	3.04 (1.86–4.97)	<0.001	2.46 (1.45–4.18)	<0.001	0.74 (0.54–1.02)^c^	0.069	1.16 (0.53–2.52)	0.71
Breastfeeding	8.81 (3.8–20.3)	<0.001	4.84 (1.87–12.5)	0.001	2.85 (1.90–4.26)^c^	<0.001	1.75 (0.77–3.96)	0.18
Male Sex	1.57 (1.01–2.45)	0.046	1.60 (1.02–2.52)	0.042	1.29 (0.93–1.86)	0.125	…	…
Maternal personal income<$100/month	5.45 (1.98–15.0)	0.001	5.30 (1.92–14.6)	0.001	0.94 (0.63–1.41)	0.776	…	…
Assignment to Infant Zidovudine1 to 6 months	1.02 (0.62–1.66)	0.94	…	…	3.08 (2.22–4.28)	<0.001	2.82 (1.27–6.29)	0.011
Shared or No Toilet/Latrine	1.21 (0.67–2.17)	0.53	…	…	0.56 (0.32–0.99)	0.045	0.54 (0.30–0.97)	0.034
Maternal Education, Secondaryor More	0.96 (0.58–1.58)	0.87	…	…	0.61 (0.41–0.93)	0.020	0.63 (0.41–0.96)	0.0334
Village residence	0.86 (0.56–1.32)	0.49	…	…	1.41 (1.02–1.95)	0.039	1.42 (1.02–1.99)	0.040
Maternal CD4+cell count (per100 cells/ µL)	0.98 (0.88–1.09)	0.73	…	…	1.02 (0.94–1.10)	0.64	…	…
Maternal Age (per 10 years)	0.83 (0.56–1.24)	0.36	…	…	0.90 (0.68–1.21)	0.496	…	…
Small for Gestational Age(<10th percentile)	1.39 (0.77–2.51)	0.28	…	…	1.22 (0.77–1.94)	0.39	…	…
Premature (<37 weeks gestation)	1.33 (0.75–2.25)	0.34	…	…	1.03 (0.95–1.11)	0.54	…	…

Prophylactic cotrimoxazole, maternal antenatal HAART use, and infant feeding method are included in the multivariable model, as are other significant factors from the univariate analysis.

OR, odds ratio; aOR, adjusted odds ratio; CI, confidence interval; HAART, highly-active antiretroviral therapy.

a Wald chi-square.

b To avoid confounding effect of infant feeding method, univariate estimate for effect of cotrimoxazole is restricted to formula-fed infants. Multivariable analysis includes both formula-fed and breastfed infants.

c A modest but significant interaction was noted between feeding method and maternal HAART with increased risk of severe neutropenia associated with breastfeeding from a mother receiving HAART. However, in multivariable analysis this interaction was no longer significant.

Recovery of hemoglobin concentrations from the expected physiologic nadir between 6 and 8 weeks [23] did not appear to be adversely affected by cotrimoxazole in formula-fed infants. Mean hemoglobin for formula-fed infants receiving cotrimoxazole measured at 6 months was slightly higher than that of infants not receiving cotrimoxazole measured at 7 months, 11.9 versus 11.6 g/dL, respectively (*P* = 0.010).

#### Severe neutropenia

Fourteen CTX infants (6.9%) and 150 CTX-unexposed infants (10.0%) developed severe neutropenia. These events were also clustered among breastfed infants (133 of 154 severe neutropenia incidents occurred in breastfed infants). Stratifying by infant feeding method, CTX was not significantly associated with severe neutropenia, OR 1.41 (0.73 to 2.69), P = 0.29. The estimated confidence interval of the risk difference (2.0%, 95% CI −1.3 to 5.2%) indicates that an absolute increase in the proportion with severe neutropenia associated with cotrimoxazole could be as large as 5%.

After adjustment for infant feeding method, antenatal maternal HAART, and significant univariate factors, the adjusted odds ratio for severe neutropenia for CTX versus CTX-unexposed was 0.80 (95% CI 0.33 to 1.93). Prolonged infant zidovudine exposure (birth to 6 months in Mashi breastfed infants), village residence, and measures of decreased socioeconomic measures were associated with increased risk of severe neutropenia in the multivariable model.

## Discussion

HIV-exposed uninfected (and predominantly formula-fed) infants who received cotrimoxazole prophylaxis from 1 through 6 months of age experienced low frequency of severe anemia or neutropenia during this period (1.1% and 5.9% of infants, respectively). Through comparison with historic and contemporaneous infant cohorts, we found that that HIV-exposed uninfected infants assigned to cotrimoxazole prophylaxis from 1 to 6 months of age did not have significantly elevated risk for severe anemia or neutropenia. Rather, maternal HAART and breastfeeding were important factors associated with increased risk of severe hematologic events.

Despite reluctance among clinicians to use cotrimoxazole in patients at risk for anemia or neutropenia, the contribution of prophylactic cotrimoxazole to these risks has not been previously assessed in HIV-EU infants. HIV-exposed infants with anemia appear to be at increased risk of death [28] and anemic children, at least those with iron deficiency, [29] have poorer neurocognitive outcomes. In a recent analysis of data from HPTN-046 conducted in Uganda and Zimbabwe, nearly half of breastfed HIV-EU infants exposed to prolonged cotrimoxazole developed severe anemia or neutropenia by 6 months of age. [30] However, as all infants were given cotrimoxazole in that study, the analysis was unable assess whether cotrimoxazole contributed to the high observed frequency of severe anemia or neutropenia.

It is reassuring therefore, that prolonged cotrimoxazole prophylaxis was not significantly associated with increased risk of severe anemia in the current study. While the design cannot exclude the possibility of association, the magnitude of any increased risk of severe anemia is unlikely to be of clinical significance. The upper bound of the 95% confidence interval was less than 1% absolute increase in frequency.

The clinical importance of asymptomatic neutropenia is uncertain, particularly when normative ranges for African infants may be significantly lower than ranges in the populations used in the development of the DAIDS toxicity tables used in this and other studies. [31]–[33] However, the findings of this study indicate that an absolute increase in severe neutropenia related to cotrimoxazole is likely to be at most 5%. In settings where either continued breastfeeding or limited access to early infant HIV diagnosis make the use of cotrimoxazole prophylaxis a consideration, these negative findings are important to inform decisions about the optimal period for prophylaxis.

This analysis was subject to several limitations. With the majority of HIV-infected women in Botswana opting to formula-feed their infants during the study period, we were unable to enroll sufficient number of breastfeeding infants to fully assess whether cotrimoxazole is associated with severe hematologic toxicity in breastfed infants, who are at greater risk of anemia. While harmonized data collection across the study cohorts facilitated adjustment for potential confounding, some residual confounding is likely given differences in the design of the parent studies and possible temporal effects. Additionally, the observed low event frequency in the CTX cohort makes multivariable adjustment and calculation of confidence limits challenging. Despite these limitations, the findings indicate that a clinically significant impact of cotrimoxazole on severe hematologic toxicity is unlikely.

In summary, we found that prophylactic cotrimoxazole in HIV-exposed uninfected infants was not associated with an increased risk of severe anemia or of severe neutropenia, even in the setting of maternal HAART use. Concerns related to possible hematologic toxicity should not limit the use of prophylactic cotrimoxazole among HIV-exposed infants.

## Supporting Information

Table S1 **Comparison of Study Characteristics.** Note: CTX, cotrimoxazole; ULN, upper limit of normal; AST, aspartate aminotransferase; ALT, alanine aminotransferase; HAART, highly-active antiretroviral therapy; ZDV, zidovudine; 3TC, lamivudine; NVP, nevirapine; ABC, abacavir; LPV/r, ritonavir-boosted lopinavir; PCR; polymerase chain reaction; SMX/TMP, sulfamethoxazole/trimethoprim.^ a^Mothers were required to be on HAART at time of delivery, consequently had met national criteria for HAART initiation previously (nadir CD4≤250 cells/ µL or AIDS). But any current CD4 count was permitted.^ b^Recommended and most common antenatal regimen. Some women received alternative regimens.^ c^Dose of infant zidovudine varied by infant age: birth to 1 month (4 mg/kg twice daily), 1 to 2 months (4 mg/kg three times daily), and 2 to 6 months (6 mg/kg three times daily).^ d^In August 2002 protocol was amended to provide single-dose nevirapine to all infants.^ e^Infants randomized to breastfeeding and long zidovudine group, had additional measurements at 2, 3, 5, and 6 months.
(DOCX)

Checklist S1 **CONSORT checklist.**
(DOC)

Protocol S1 Study protocol “Safety of cotrimoxazole prophylaxis in HIV- and HAART-exposed infants in Botswana”.
(PDF)
