# Supplementary material for: Cotrimoxazole Prophylaxis and Risk of Severe Anemia or Severe Neutropenia in HAART-Exposed, HIV-Uninfected Infants
Source: PLoS One. 2013 Sep 23;8(9):e74171. doi: 10.1371/journal.pone.0074171 (PMC3781096; doi:10.1371/journal.pone.0074171)
Supplement: Table S1 — Comparison of Study Characteristics. Note: CTX, cotrimoxazole; ULN, upper limit of normal; AST, aspartate aminotransferase; ALT, alanine aminotransferase; HAART, highly-active antiretroviral therapy; ZDV, zidovudine; 3TC, lamivudine; NVP, nevirapine; ABC, abacavir; LPV/r, ritonavir-boosted lopinavir; PCR; polymerase chain reaction; SMX/TMP, sulfamethoxazole/trimethoprim. aMothers were required to be on HAART at time of delivery, consequently had met national criteria for HAART initiation previously (nadir CD4≤250 cells/ µL or AIDS). But any current CD4 count was permitted. bRecommended and most common antenatal regimen. Some women received alternative regimens. cDose of infant zidovudine varied by infant age: birth to 1 month (4 mg/kg twice daily), 1 to 2 months (4 mg/kg three times daily), and 2 to 6 months (6 mg/kg three times daily). dIn August 2002 protocol was amended to provide single-dose nevirapine to all infants. eInfants randomized to breastfeeding and long zidovudine group, had additional measurements at 2, 3, 5, and 6 months. (DOCX) [file pone.0074171.s001.docx]

|  |  | **Mashi**  **(CTX-unexposed)** | **Mma Bana**  **(CTX-unexposed)** | **CTX Safety**  **(CTX)** |
| --- | --- | --- | --- | --- |
| **Total Infant Enrollment** (current analysis) | | 1190 (930) | 709 (636) | 257 (209) |
| **Years of Enrollment** | | 2001-2003 | 2006-2008 | 2009-2010 |
| **Enrollment Criteria** | |  |  |  |
|  | Time of Enrollment | 33-35 weeks gestation | 18-34 weeks gestation | Birth |
|  | Site | Lobatse, Gaborone, Mochudi, Molepolole | Lobatse, Gaborone, Mochudi, Molepolole | Gaborone, Molepolole |
|  | Maternal Age | ≥18 years | ≥18 years | ≥21 years |
|  | Infant Feeding | Willing to breastfeed or formula-feed | Willing to breastfeed | Either breastfeed or formula-feed |
|  | Maternal Hemoglobin | ≥8 g/dL | ≥8 g/dL | Any |
|  | Maternal CD4 | Any | Any | Any ^a^ |
|  | Maternal Absolute Neutrophil Count | ≥ 1000 cells/μL | ≥ 1000 cells/μL | Any |
|  | Maternal ALT/AST | < 2.5 x ULN | < 10 x ULN | Any |
| **Maternal Treatment and Interventions** | |  |  |  |
|  | Antenatal antiretroviral therapy | Zidovudine twice daily starting at 34 weeks. After October 2002, women with CD4 ≤ 200 cells/μL: ZDV/3TC/NVP ^b^ | CD4 ≤ 200 cells/μL:  ZDV/3TC/NVP. Current CD4 > 200 cells/μL: Randomized to ZDV/3TC/ABC or ZDV/3TC/LPV/r | CD4 ≤ 250 cells/μL:  AZT/3TC/NVP ^b^ |
|  | Peripartum zidovudine | During delivery | During delivery | During delivery |
|  | Single-dose Nevirapine | Randomized to nevirapine or placebo in labor | No | No |
|  | Antenatal Iron Supplementation | Yes | Yes | Yes |
|  | Preferred Mode of Delivery | Vaginal | Vaginal | Vaginal |
|  | Standard Timing of Cord Camping | Immediate | Immediate | Immediate |
| **Infant Treatment and Interventions** | |  |  |  |
|  | Zidovudine (4 mg/kg twice daily) | Randomized to zidovudine from birth through 1 month (formula-feeding group) or birth through 6 months (breastfeeding group) ^c^ | Birth through 1 month | Birth through 1 month |
|  | Single-dose Nevirapine (6 mg once) | Randomized between nevirapine and placebo ^d^ | Yes | Yes |
|  | HAART for HIV-Infected | Yes | Yes | Yes |
|  | Feeding Method | Randomized to iron-supplemented formula-feeding or breastfeeding (encouraged exclusive to 6 months) | Breastfeeding (encouraged exclusive to 6 months) | Breastfeeding (encouraged exclusive to 6 months) or iron-supplemented formula-feeding |
|  | Hematologic Monitoring | Birth and 1, 4, 7 months ^e^ | Birth and 1, 3, 6 months | 1, 3, 6 months |
|  | HIV DNA PCR | Birth and 1, 4, 7 months | Birth and 1, 3, 6 months | 1, 3, 6 months |
|  | Cotrimoxazole Prophylaxis for HIV-uninfected infants (< 5 kg, 100/20 mg SMX/TMP once daily; ≥ 5 kg, 200/40 mg SMX/TMP once daily) | No | No | Yes |
