# Supplementary material for: Cotrimoxazole Prophylaxis and Risk of Severe Anemia or Severe Neutropenia in HAART-Exposed, HIV-Uninfected Infants
Source: PLoS One. 2013 Sep 23;8(9):e74171. doi: 10.1371/journal.pone.0074171 (PMC3781096; doi:10.1371/journal.pone.0074171)
Supplement: Protocol S1 Study protocol — “Safety of cotrimoxazole prophylaxis in HIV- and HAART-exposed infants in Botswana”. (PDF) [file pone.0074171.s003.pdf]

Safety of cotrimoxazole prophylaxis in HIV- and HAART-exposed infants in Botswana

A pilot study

Botswana-Harvard School of Public Health AIDS Initiative Partnership (BHP)
Private Bag BO 320, Bontleng
Gaborone, Botswana
Tel: 3902671, Fax: 3901284

1 Study sites and management

1.1 Study sites (BHP)

Gaborone, Molepolole (and if needed, Lobatse and Mochudi)

1.2 Management

All questions concerning this protocol, including issues regarding: Inclusion/exclusion criteria, clinical medical management, toxicity management, schedule of events, laboratory tests, form completion, drug availability, dose, or delinquencies should be sent via e-mail to slpeterson@partners.org. When appropriate, inquiries should include the protocol name, BHP-ID, and a brief relevant history.

2 Glossary

AE	adverse events
ART	antiretroviral therapy or treatment
BHP	Botswana-Harvard Partnership
BNP	Botswana National Programme
CRF	case report form
CTX	Cotrimoxazole
DAIDS	NIH Division of AIDS
FDA	Food and Drug Administration
FBC	Full blood count
HAART	highly active antiretroviral therapy or treatment
IRB	institutional review board
MTCT	mother to child transmission
NNRTI	nonnucleoside reverse transcriptase inhibitor
NRTI	nucleoside reverse transcriptase inhibitor
PCP	Pneumocystis pneumonia
PCR	polymerase chain reaction
PI	protease inhibitor
PMTCT	Prevention of mother to child transmission
SD	single dose
WHO	World Health Organization
ZDV	zidovudine

3 Abstract/Schema

TITLE	Safety of cotrimoxazole prophylaxis in HIV- and HAART-exposed infants in Botswana
OBJECTIVE	The primary aim is to compare the incidence of severe/life-threatening anemia occurring from 1 to 6 months of life between infants who do receive cotrimoxazole (CTX) and infants who did not receive CTX prophylaxis, among a group of children who were all exposed to maternal HIV and maternal highly-active antiretroviral therapy (HAART). The study also seeks to identify factors associated with increased early mortality/morbidity in a programmatic

	cohort of HIV-exposed, predominantly formula-feeding infants.
DESIGN	Prospectively-followed cohorts, compared with historical cohort
METHODS	Cohort A (CTX prophylaxis provided to 6 months of age by the study): infants born to HIV-infected mothers and exposed to HAART *in utero* will be recruited at birth, and will include both breastfeeding and formula-feeding infants. Baseline hematologic and HIV testing, and medical history/physical examinations will be performed birth and at 4 weeks of age prior to initiating cotrimoxazole (CTX) prophylaxis. Follow-up examinations will occur at 3 and 6 months of age. Infants will continue to take CTX until 6 months of age or until the period of HIV exposure (through breastfeeding) has ended, whichever occurs later. Rates of hematologic toxicity (anemia and neutropenia) will be compared between this cohort and the existing Botswana/BHP Mma Bana PMTCT study<br><br>Cohort B (observational cohort health outcomes): infants will be enrolled at birth, and health outcomes will be ascertained prospectively at 1 and 6 months of age. Children in cohort B will not receive CTX from the study clinic.
DURATION	Infants will be followed until 6 months of age in the study. Study expected to be completed in 18 months.
SAMPLE SIZE	Up to 222 infants will enrolled in cohort A and an additional 222 infants in cohort B.
POPULATION	Infants born to HIV-infected mothers in Botswana.

4 Specific Aims and Hypotheses

Each year, more than 2 million children are born to HIV-infected women.[1] The World Health Organization (WHO) recommends that these infants receive cotrimoxazole (CTX) prophylaxis starting at 4-6 weeks of age until the period of infant HIV transmission risk is over, and the infant is known to be HIV-uninfected[2]. There is also increasing interest in studying CTX prophylaxis given to *all* infants of HIV-infected women at the time of initiation of replacement feeding, regardless of infant HIV infection status, to mitigate the high risk of infant morbidity and mortality associated with formula feeding in the developing world. However, infant *in utero* exposure to maternal antiretroviral drugs can lead to hematologic toxicities in infants[3-5], and we are seeing moderate rates of severe anemia in preliminary data from infants exposed to maternal highly-active antiretroviral therapy (HAART) in an ongoing trial (the Mma Bana study) in Botswana.

It is critical to know whether infant CTX prophylaxis exacerbates the hematologic toxicity associated with perinatal ARV exposure. This question, with broad public health implications, has never been studied.

We therefore wish to study the hematologic toxicity associated with CTX prophylaxis given to infants exposed to maternal HAART in Botswana. We will use existing data from a large cohort that did not receive CTX, and enroll a smaller cohort that does receive CTX.

4.1 Primary Objectives:

- Compare the incidence of severe/life-threatening anemia (as defined by DAIDS toxicity table) occurring from 1 to 6 months of life between HAART-exposed infants who either receive CTX (in the new cohort) or who do not receive CTX (in the Mma Bana PMTCT cohort). [*hypothesis: CTX will increase incidence of severe or life-threatening anemia among HAART-exposed, breastfeeding infants*]

- Prospectively determine factors associated with increased risk of hospitalization or death during first six months of life in HIV-exposed, predominantly formula-fed infants. [*hypothesis: factors can be identified that distinguish infants at high-risk if formula-fed]*

4.2 Secondary Objectives:

- Compare the incidence of severe/life-threatening neutropenia (as defined by DAIDS toxicity table) occurring from 1 to 6 months of life between HAART-exposed breastfeeding infants who receive CTX or who do not receive CTX in the same cohorts [*hypothesis: CTX will increase incidence of neutropenia among HAART-exposed, breastfeeding infants*]

- Determine the rates of severe/life-threatening anemia and neutropenia (as defined by DAIDS toxicity table) occurring from 1 to 6 months of life among *formula fed* HAART-exposed infants who receive CTX [*hypothesis: CTX will be associated with high rates of anemia/neutropenia following in utero HAART exposure, even in the absence of ARV exposure through breast milk; these toxicities may be exacerbated among children whose mothers were on HAART at time of conception, compared with children whose mothers started HAART later in pregnancy]*

- In an exploratory analysis, compare infant morbidity, mortality and hospitalizations occurring between 1 and 6 months of life between the new cohort of CTX-exposed infants and the existing (Mma Bana and Mashi) cohorts of CTX-non-exposed infants [*hypothesis: CTX may appear protective, but larger numbers will be needed to evaluate this question*]

- Compare the prevalence of iron, vitamin B12, and folate deficiency in infants with severe/life-threatening anemia (as defined by DAIDS toxicity table) occurring from 1 to 6 months of life with the prevalence in a subset of infants without anemia. [*hypothesis: deficiencies will be common in babies with anemia*]

- Assess maternal knowledge regarding chosen infant feeding method (and counseling received); explore potential predictors of infant mortality, by feeding strategy.

5 Background and Rationale

5.1 Overview

Botswana and other middle-income and developing countries are scaling up HAART for HIV-infected pregnant mothers in accordance with WHO guidelines.[6] It is hoped that greater virologic suppression during pregnancy will lead to improved maternal and infant outcomes. Concurrently, urged by the WHO, national programs have pushed to make cotrimoxazole prophylaxis available to HIV-exposed infants for prevention of early opportunistic infection. However, both cotrimoxazole (CTX)[7] and important components of HAART[8-10] are known to cause anemia and neutropenia. The potential additive toxicity of these therapies has not been systematically studied in infants in a developing world context.

5.2 Early childhood anemia

Childhood anemia is common in the developing world, found in more than 50% of children under age of 4.[11] Infants with mild or moderate anemia related to iron deficiency have impaired physical and cognitive development.[12, 13] Severe anemia alone is a leading cause of hospitalization and death in the developing world.[14] In addition, severe anemia in infants has been demonstrated in several settings to be an independent predictor of mortality during treatment for other conditions.[15-17] While prevention efforts have largely focused on malaria control and iron supplementation, the causes of anemia in most sub-Saharan African countries is more complex. A recent in depth study of the etiology of severe anemia in Malawian children, found that bacteremia, vitamin B12 deficiency, HIV, parasitic infection, and G6PD genetic disorder were the most important contributors to life-threatening anemia.[18] In countries with prevalent anemia, prophylactic treatment with CTX could serve to reduce anemia by preventing bacteremia or severity of malaria. However, it could also worsen anemia by adding further exacerbating vitamin B12 deficiency megaloblastic anemia. Furthermore, the interaction between these conditions and cotrimoxazole and/or HAART is unknown. In Botswana, as in many developing countries, blood transfusion or other acute therapies for anemia are not readily available, making the consequences of severe anemia in these children even more concerning. Therefore, the hematologic toxicity of medications should be determined and carefully weighed against their benefits.

5.3 Infant anemia and maternal HAART

Public clinics and hospitals in Botswana are providing HAART to pregnant, HIV-infected women with CD4+ cell count < 250 cells/mm[3,19] Maternal HAART has been shown to reduce both maternal mortality as well as maternal to child transmission (MTCT) of HIV.[20] While *in utero* exposure to HAART has been associated with pediatric anemia and neutropenia in the developed world,[3-5] these abnormalities generally have not been severe and have appeared to resolve with time in studies from this setting. However, in an ongoing study of maternal HAART during pregnancy and breastfeeding in Botswana (the "Mma Bana" study) that is enrolling 750 pregnant HIV-infected women, a preliminary review of safety data suggests approximately 6-10% of infants with sufficient follow-up thus far have developed a new grade 3 (severe) or 4 (life-threatening) anemia between 1 and 6 months of age (Botswana-Harvard Partnership, unpublished data, 2007). HIV-uninfected infants in the Mma Bana study do not receive CTX prophylaxis, as they are tested frequently in real-time for HIV (and CTX is started as soon as a diagnosis of HIV is made), and because of concern regarding overlapping hematologic toxicity between maternal HAART exposure and infant CTX. Somewhat lower rates of anemia were seen in the prior Mashi PMTCT study that was conducted at the same sites and in which infant CXT prophylaxis as not given, for the same reasons[21]. Anemia in the Mma Bana study may in part be related to increased exposure to

antiretroviral medications *in utero* and possibly to low-level exposure through breast milk.[22]

5.4 Infant cotrimoxazole prophylaxis

We are concerned that the anemia in HAART-exposed infants could be exacerbated by the addition of CTX. Prophylactic CTX is recommended by the WHO[2] starting at 4-6 weeks of life and continuing until the infant is known to be HIV-uninfected and is no longer at risk for HIV infection through breastfeeding, to prevent *Pneumocystisis jirovecii* pneumonia (PCP) and invasive bacterial infections, for which HIV-infected infants are at high risk.[23, 24] MTCT rates among infants born to HIV-infected women who take HAART during pregnancy and breastfeeding may be as low as 2-4%; this low MTCT rate may affect the decision about whether to routine CTX prophylaxis in such instances, depending on the safety of infant CTX prophylaxis. As recommended, Botswana provides breastfeeding HIV-exposed infants with daily CTX prophylaxis. However, due to challenges associated with early infant diagnostic testing (HIV-1 DNA PCR), even formula-fed infants of HIV-infected women often receive CTX for 6 months or more until it can be determined that they are uninfected (in Botswana and in most resource-limited settings).

Additionally, CTX prophylaxis may soon be considered for a wider population. Infants in sub-Saharan African countries are at high risk for morbidity and mortality related to infectious diseases (particularly gastroenteritis and pneumonia) at the time of weaning or if breastfeeding is not initiated.[25-27] HIV-exposed, formula-fed infants are at particularly high risk.[21] There is increasing interest in studying CTX prophylaxis in a randomized controlled trial among HIV-exposed (predominantly HIV-uninfected) infants, particularly at the time of weaning from breastfeeding, to decrease this morbidity and mortality. This strategy is supported by the findings from a Ugandan study that found a 63% reduction in morality among HIV-uninfected children when an HIV-infected household member took CTX prophylaxis.[28]

5.5 Hematologic toxicity of cotrimoxazole

Cotrimoxazole can adversely affect hematopoiesis, although virtually no data are available on this in infants.[7] In studies of prophylactic treatment in patients receiving myelosuppressive chemotherapy, CTX appears to worsen severity and slow recovery from pancytopenia.[29, 30] Similarly, there are reports of increased hematologic toxicity of CTX with concurrent HAART in sub-Saharan adults.[10, 31, 32] This effect may be intensified in infants, as infants appear to be more vulnerable to the hematologic toxicity associated with antiretrovirals.[33] Provision of CTX prophylaxis to young infants may potentiate already significant anemia and neutropenia in HAART-exposed infants. The potential contribution of comorbid conditions— vitamin B12 and iron deficiencies, malnutrition, bacteremia, and malaria— should not be underestimated.

Conversely, provision of CTX could be protective against prevalent anemia. As detailed above, malaria and bacteremia appear to account for many episodes of severe anemia in sub-Saharan Africa.[18] CTX may lower prevalence of both of these infections and thus, in fact, prevent anemia.

There is no existing literature evaluating the safety of CTX in HAART-exposed, but largely HIV-uninfected children.

5.6 Morbidity and Mortality with Replacement feeding

Provision of antiretroviral therapy has nearly eliminated peripartum transmission of HIV to infants in resource-rich countries[34] and in resource-limited settings where therapy is available[35]. However, transmission via breastmilk accounts the majority of cases in

areas where peripartum therapy is available[36]. Breast milk transmission in resource-limited settings erodes the gains made through improved access to services for prevention of peripartum MTCT.

Replacement feeding with infant formula can eliminate this risk, however clinical trials of replacement feeding have had mixed results in regions of high HIV prevalence. In a randomized trial in Botswana, HIV-exposed infants randomized to replacement feeding (vs. breastfeeding with extended prophylaxis) had significantly less risk of HIV-infection but were more likely to die by six months of life than infants who were breastfed. In an observational South African cohort, mortality was nearly three times higher in infants who were fed with formula, 15.1 versus 6.1 percent[37]. In both cohorts, contamination of formula did not seem to lead to the increased mortality in formula-feeding infants, but rather a broader increase in pneumonias, severe gastroenteritis, and sepsis. However, in studies in Kenya and Côte d'Ivoire formula-feeding appeared safe. In these in urban cohorts with access to municipal water, mortality was high, but similar between breastfed and formula-fed infants[38, 39].

These conflicting data suggest that the safety of replacement feeding depends on local factors and likely differs between regions and populations. Safety may depend on prompt self-referral to basic medical care in cases of infant illness or upon other factors. Each of the studies noted above was conducted in populations with safe, treated water, intensive education on preparation of infant formula, and good access to study clinicians. Outcomes in a programmatic setting may well differ from those in a clinical trials setting, which further heightens the importance of assessing— in a program setting— actual outcomes and predictors of adverse outcomes among formula-fed children. Remarkably, this type of evaluation has rarely been performed.

5.7 Predictors of Increased Risk of Replacement Feeding

The WHO and UNICEF have long recommended replacement feeding for infants born to HIV-infected mothers as long as it is acceptable, feasible, affordable, safe, sustainable (AFASS).[40] The suggested criteria are largely related to safe water supply and formula preparation, and their utility is unproven. More than 80 percent HIV-infected mothers in Botswana formula-feed their babies.[35] National guidelines advise counselors to use the AFASS criteria.[41] These criteria may or may not identify which infants can safely formula-feed and have proven difficult to implement.[42-44] Improved, simple infant feeding guidelines are needed to assist practitioners in resource-limited settings.

Little research has been published to evaluate individual or groups of risk factors that may be associated with morbidity or mortality with formula feeding. Using data drawn from several low-income countries, researchers at the WHO found in a predominantly HIV-unexposed population of infants that risk of poor outcome was associated low maternal education level.[45] In a Ugandan HIV-exposed cohort, low socioeconomic status, advanced maternal immunosuppression, and low birth weight was associated with early mortality in formula-fed infants.[46] In South Africa, researchers modified the AFASS criteria and found that HIV-free survival was associated with having piped water, using electricity, kerosene, or gas for fuel, and having disclosed HIV status to household. They found that formula-fed infants in households that did not meet all three criteria were at greater than three-fold increased risk of death or HIV infection.[43] Preliminary data from our group in Botswana on predictors of early mortality is described below.

Despite its great public health importance, we do not know how to determine whether it is safe for an individual HIV-infected mother to formula-feed her baby, nor how to most effectively mitigate the excess risk of early mortality in HIV-exposed, but largely uninfected infants. With a preponderance of evidence supporting the use of replacement

feeding to avoid HIV acquisition, the vast majority of HIV-infected women in Botswana are advised to use infant formula. We hope to document the general safety of formula in a program setting in one urban area and one village in Botswana—something that also has not been systematically attempted.

However, the risks associated with formula feeding for *some infants* may exceed the benefits. We urgently need methods to ascertain what constitutes "safe" in AFASS for an individual baby in a program setting – this is the second aim of this study. Knowledge of these factors would help caregivers identify infants at high risk for adverse outcome with formula feeding, and target them for supported breastfeeding, or for other potentially-effective interventions in the context of formula feeding, such as enhanced home health outreach, or possibly continued cotrimoxazole prophylaxis.

5.8 Summary

The question of CTX prophylaxis safety (particularly following exposure to maternal HAART) and predictors of risk in formula-feeding infants is important for three principal reasons:

- Potential effect on WHO recommendations for infant CTX prophylaxis: high rates of hematologic toxicity associated with CTX prophylaxis (particularly in HAART-exposed infants) might alter the risk-benefit considerations behind these recommendations, particularly if the risk of late infant HIV infection decreases significantly with maternal HAART during breastfeeding, as seems to be the case from early reports.[47, 48]

- Use of CTX to decrease infant morbidity and mortality at the time of weaning from breastfeeding or in targeted high-risk formula-fed infants: if CTX in HAART-exposed, formula-fed infants is found to be safe, this would help inform the design of a randomized controlled trial of CTX prophylaxis to prevent excess morbidity and mortality in HIV-exposed infants at the time of breastfeeding cessation and formula initiation.

- Help inform counseling and feeding recommendations for HIV-infected mothers.

6 Preliminary Studies

We have a unique opportunity to evaluate the safety of CTX in Botswana, building upon large and closely-monitored existing cohorts of HIV-exposed children who did not receive CTX prophylaxis. CTX prophylaxis was not offered to HIV-negative infants in these studies due to early and frequent real-time study-related infant HIV testing and concerns regarding overlapping toxicity with infant ZDV and maternal HAART exposure during breastfeeding (although CTX was and is started as soon as an infant is diagnosed as being HIV infected).

6.1 Mashi study

The Mashi study[21, 49] recruited 1200 HIV-infected pregnant women at 4 sites in Botswana between 2001 and 2003. All women received zidovudine (ZDV) from 34 weeks gestation, and infants were randomized to either formula feed (with 1 month of infant ZDV) or to breastfeed (with 6 months of infant ZDV). Mothers and infants were randomized to receive a single dose of nevirapine (NVP) or placebo. HAART became available in Botswana during the Mashi study and 71 women with CD4 less than 200 started 3-drug HAART antepartum. HIV-negative infants were not given routine CTX, as they were tested at short intervals for HIV and were started on prophylaxis only if they

became HIV-infected. Formula-feeding was found to be protective against HIV infection, but excess infant mortality was in seen these children and in breast-fed infants at time of weaning.[21]

A preliminary analysis of factors affecting mortality has been presented,[50] with infant HIV infection, presence of a household latrine, low birth weight, and formula-feeding associated with mortality by two years of life. However, Mashi was not designed to evaluate detailed risk factors for adverse infant outcomes with formula feeding, and formula was also offered as an intervention in a rigorous clinical trial with 8 study visits in the first month of life.

Eleven infants (~2%) developed severe or life-threatening anemia during follow-up.

6.2 Mma Bana study

The Mma Bana study is recruiting 750 pregnant HIV-infected women from the same 4 sites in Botswana. Women with low CD4+ receive lamivudine (3TC), ZDV, and NVP antepartum and indefinitely (n=170), and women with higher CD4+ (who do not require HAART for their own health, but are receiving it for PMTCT) are randomized to 3TC, ZDV, and abacavir or 3TC, ZDV, and lopinavir/ritonavir in pregnancy and during 6 months of breastfeeding (n=560). The primary objectives of the Mma Bana trial are to determine the safety and efficacy of different maternal HAART regimens in preventing overall MTCT as well as MTCT related to breastfeeding; it is the first randomized trial of HAART regimens for MTCT prevention to ever be conducted.

Similar to the Mashi study, the infants of the Mma Bana studies are followed closely, with very low loss to follow-up rates. Visit schedules are almost identical, with HIV DNA PCR as well as hematology (CBC) checked at delivery, 1, 3-4, and 6 months. Children do not receive CTX prophylaxis unless they were diagnosed as HIV-infected, for reasons noted above. Relevant data (see below) from these studies will be available.

However, a somewhat higher rate of infant anemia has been observed in interim analysis. Approximately 6-10% of infants with sufficient follow-up thus far have developed a new grade 3 (severe) or 4 (life-threatening) anemia between 1 and 6 months of age. This may be related to increased infant exposure to nucleoside analogs both in utero and via breast-milk. Further analysis is planned.

7 Study Design and Conduct

7.1 Overview

In cohort A, we will evaluate the safety of prophylactic daily CTX administered between 1 and 6 months of age in a new recruited cohort of HIV and HAART-exposed infants. We will compare the hematologic parameters in these children with those in the existing cohorts of infants born to women with CD4 counts < 250 cells/mm^3 from the Mma Bana and Mashi studies. Children will be recruited to the new cohort from the maternity wards of the same district hospitals as used in the Mma Bana and Mashi studies. Both breastfed and formula fed infants will be enrolled and receive CTX prophylaxis. All infants will receive ARV prophylaxis as per Botswana national PMTCT guidelines (currently, single-dose NVP and 4 weeks of ZDV prophylaxis).

In cohort B, we will evaluate possible baseline predictors of risk of infant hospitalization and death between birth and 6 months of age among predominantly formula-fed infants born to HIV-infected women. Infants in cohort B will receive all care from government clinics (and will not receive CTX through the study clinic). Infants will be followed at 1 and 6 months of age to ascertain clinical outcomes.

7.2 Inclusion criteria- Mothers

- Documented HIV infection (by positive ELISA or rapid HIV-1 test)
- Cohort A: taking 3-drug HAART at any point during current pregnancy (note: can include 2 NRTI+NNRTI, 2NRTI+PI, or 3 NRTI)
    - Cohort B: No restrictions on maternal ARVs
- 21 years of age or older, and able and willing to sign informed consent.
- Proof of Botswana Citizenship (required by the government ARV and PMTCT programs)

In order for a mother to be eligible under the last criterion, she must display their Omang, Botswana passport, or application for Omang (new or renewal). A copy of this proof of citizenship is kept in the participant's clinical record.

7.3 Exclusion criteria- Mothers

- Involuntary incarceration.

7.4 Inclusion criteria- Infants

- Younger than 42 days of age
- Able to be brought to regular visits at study clinic until at least 6 months postpartum

7.5 Exclusion criteria- Infants

- Known pre-existing birth anomalies resulting in a high probability that the baby will not survive to 6 months
- Cohort A: Known hypersensitivity to cotrimoxazole

Note that children can participate in both Cohorts A and B regardless of the feeding method that the mother has chosen.

7.6 Recruitment

Post-partum women will be recruited from among women delivering liveborn infants in Gaborone (principally Princess Marina Hospital) and in Molepolole (principally Scottish Livingstone Hospital). Depending on the pace of recruitment, participants may also be recruited from the maternity wards at Athlone Hospital in Lobatse and Deborah Retief Memorial Hospital in Mochudi.

In collaboration with health clinic staff, members of the study team will facilitate maternal antenatal CD4 count testing, timely return of CD4 results, and initiation of HAART in women eligible according to the national program guidelines. Members of the study team will, with the agreement of health clinic staff, provide health education presentations/health talks in the referring antenatal clinics. These presentations will focus on the national guideline recommendations for the use of cotrimoxazole. The study will be introduced and described. Interested women who are potential study candidates would be offered more information and encouraged to discuss participation with family or other advisors.

After delivery women who are potentially eligible will be approached regarding their potential interest in participating in this study. Generally, this will occur among postpartum women admitted to maternity wards who have delivered liveborn infants. However, in some circumstances women may be approached after discharge during their post-partum clinic visit. Study staff (recruiters, nurses, and physician[s]) will liaise with the nurses and physicians of the maternity wards to identify potential study participants and establish a time to approach patients that is agreeable to patients and hospital/clinic staff.

7.7 Consent

Women and their infants who are potentially eligible to participate in the study will be provided in-depth information regarding the benefits and risks of the study as well as information about the Botswana PMTCT Program. Participants will review all of the information contained in the Informed Consent form in depth with study staff, and will be encouraged to ask questions and to discuss the study in detail. Mothers of infants who are potentially eligible to participate in Cohort A may decide to consent to participating in either Cohort A or Cohort B. Mothers of infants who are not eligible for Cohort A (i.e., mothers who were not on HAART during current pregnancy or infants with known allergy to CTX) may consent to participate in Cohort B.

Among women who are willing to participate and have her child participate in the study, consent will be indicated by signing and dating the Informed Consent form. After providing consent, patients will be given a signed copy of the consent form to keep.

Women who provide consent to participate for themselves and their baby will also be asked whether or not they would allow study staff to follow them and their baby in the case of a missed visit and, if so, to provide their contact information.

7.8 Participant ID (PID) Assignment

Each participant who is seen, meets the above eligibility criteria, and signs informed consent after a full discussion of the study will have a Participant ID number (PID number) assigned.

7.9 Birth (enrollment) visit

Written informed consent will be obtained from mothers if they/their infants meet eligibility criteria. Consenting mothers will indicate whether or not they agree to have their infant participate in Cohort A or Cohort B (depending on eligibility).

Study staff will gather and record baseline data from the patient and the medical record. Information to be collected for mother and infant includes: demographics/SES, attitudes toward healthcare, planned method of feeding and counseling/decision process related to chosen feeding method, medical history, current medications, HIV history, ARV use during pregnancy, maternal hemoglobin (if known), course of pregnancy, gestational age, birth weight, mode of delivery, and results of diagnostic studies.

Maternal contact information will be taken (if she consents to this), and the mother will also be given contact information for the study clinic, and an appointment for a 4-week visit at the BHP study clinic. Mothers/infants will be counseled to seek routine postnatal and well baby care (including formula provision, for formula-fed babies) through the Government health system.

7.10 Week 4 visit

Consenting, eligible women and their infants will be scheduled for a visit at the BHP study clinic four weeks (+/- 1 week) after birth. The follow-up schedule of the study will be explained again and subjects will be encouraged to ask questions.

7.10.1 Cohort A,

Events occurring since birth including infant illnesses, hospitalizations (or prolongation of initial hospitalization related to delivery), mortality, medications, health-seeking behavior, identity of baby's primary caregiver(s), duration of ZDV prophylaxis, and method of feeding will be recorded on CRF. A targeted infant physical examination will be performed. The infant will be given cotrimoxazole through the study.

An infant blood sample will drawn for baseline full blood cell count (FBC) with differential and HIV DNA PCR. If results indicate Grade 3 or 4 anemia and/or neutropenia by current DAIDS toxicity tables, participant will be contacted and asked to return for repeat FBC evaluation and treatment as applicable (within 2-4 weeks for a grade 3 anemia or grade 3 or 4 neutropenia, within 1 week for a grade 4 anemia).

It is expected that the bulk of early hematologic toxicity will be as a result of ZDV prophylaxis during first 4 weeks. Infant anemia and neutropenia are expected to resolve with greater time from this intervention. Thus, the protocol for managing anemia and neutropenia during study initiation is different than as described in Safety and Toxicity below.

If Grade 4 anemia and/or neutropenia is present on repeat draw, cotrimoxazole will be held until less than Grade 3 and asymptomatic. If infants are symptomatic with Grade 3 anemia or neutropenia, cotrimoxazole will also be held until less than Grade 3 and asymptomatic. Enrolled infants not taking CTX will continue to be followed on-study until 6mos of age using the same schedule of evaluations, if the participant's caregiver agrees to this (and these infants will contribute to Cohort B).

If infants are asymptomatic and Grade 3 anemia and/or neutropenia is present on repeat draw, cotrimoxazole will be continued. Infant will be monitored closely and will have repeat full blood counts until less than Grade 3.

If anemia or neutropenia subsequently worsens after study initiation it will be managed as described in Safety and Toxicity below.

7.10.2 Cohort B

Infants may participate in observational cohort B, either because they were not eligible for cohort A; the mother chose participation only in cohort B; or an infant in cohort A experienced a limiting anemia or neutropenia, as noted above.

The same clinical and targeted physical examination information will be collected at 4 weeks in cohorts A and B. Information regarding initiation of cotrimoxazole prophylaxis at the government clinic will also be recorded. If not previously done outside of the study clinic, a sample(s) will be collected for HIV DNA PCR in accordance with the National Program. As in cohort A, test results will be shared with mother/baby as promptly as possible, to assist with medical care/decisions.

7.11 Study Medication

Cotrimoxazole
All infants enrolled in cohort A, irrespective of HIV-status and feeding method, will be started on standard doses of daily CTX prophylaxis (less than 5kg: 100mg sulfamethoxazole, 20mg trimethoprim; greater than 5kg: 200mg sulfamethoxazole, 40mg trimethoprim). The cotrimoxazole administered will be a generic, oral solution formulation to be taken by mouth. Medication will be dispensed by study staff in increments of up to 3 month's supply and will be refilled during study visits.

After the 6-month visit, CTX will be stopped in HIV-uninfected infants without ongoing exposure to breastmilk. However, mothers who continue to breastfeed will be advised to continue infant CTX. An adequate supply of CTX to allow for transition to the government clinics will be provided. Unless a contraindication exists, the mother will be advised to continue infant CTX until the infant is weaned and known to be HIV-uninfected.

Caregivers will be counseled to continue CTX prophylaxis to all HIV-infected infants.

Note: infants in cohort B may receive CTX prophylaxis from non-study health clinics as part of routine care; this information will be captured/recorded in the study, but the study will not provide CTX prophylaxis to infants in Cohort B. Per international clinical guidelines and Botswana policy, CTX prophylaxis is discontinued in HIV-exposed infants once infant HIV infection is ruled out (and after the cessation of breastfeeding).

HAART
Mothers (and HIV-infected infants) will be referred for and will obtain anti-retroviral medications via the Government of Botswana National Program. Prescribed regimens will depend on national guidelines in place at time of treatment.

Formula
Free infant formula is available through Government clinics at which infants will be followed. Mothers/caregivers will be supported to obtain formula from these clinics for if they have chosen to formula feed. Formula will not be distributed by the study clinics.

7.12 Follow-up

Follow-up visits for mothers and infants in cohort A will occur at 1, 3 and 6 months postpartum/of age (and more frequently as needed, in cases of toxicity/infant illness). Follow-up in cohort B will occur at 1 and 6 months. Mothers will be informed of any available test/laboratory results during these visits.

Clinical Evaluations
- Assessment of new or resolved infant signs/symptoms/diagnoses or the use of any infant concomitant medications (cohort A only)
- All hospital admissions (other than for normal delivery, and to include prolongation of initial delivery-related hospitalization, due to infant illness) and deaths will be recorded along with associated diagnoses
- Collection of maternal (and for infected infants, pediatric) HAART use information
- Infant weight, length, and head circumference and targeted infant physical exam (as needed, if symptoms reported)
- Information on feeding method (and counseling regarding appropriate infant feeding, as per Botswana National PMTCT Guidelines).

Laboratory evaluations:

- Cohort A: Infant full blood count with differential and HIV-1 DNA PCR at 1, 3, and 6 months (the 3 month sample will only be tested if the 6 month PCR is positive and the 1 month PCR is negative).
- Cohort B: HIV-1 DNA PCR as per Government National Program guidelines—i.e., at 1 month, and after cessation of breastfeeding in breastfed infants, if testing not already performed through Government system. No scheduled hematologic monitoring.
- General maternal laboratory information that is available in maternal medical records (separate maternal samples will not be collected as part of this study).

7.13 <u>HIV testing</u>
Infants will have blood sample drawn for HIV-1 DNA PCR testing. Pre- and post-test counseling will be provided to the mothers.

Mothers participating in this study will all, by definition, have previously tested positive for HIV-1 infection and have received HAART for HIV-1 treatment during pregnancy. For mothers who do not have written documentation confirming their prior positive HIV test result, confirmation of HIV infection may be performed by study staff.

Any tests that are not part of routine, Botswana National Program standard of care will be funded by the study. Separate HIV testing consent will not be performed (an opt out approach will be taken, as per Botswana guidelines).

7.14 <u>Anemia studies—Cohort A only</u>
Infants in cohort A with Grade 3 or 4 anemia after baseline will have additional studies performed on their blood samples to evaluate for etiology of feeding. An equal number of matched (age, weight, feeding method) non-anemic infants will also be selected. Additional studies may include iron/iron binding capacity, vitamin B12 levels, vitamin A, blood smear, and/or folate. Number of infants selected and studies will depend on resources available; we estimate that up to ~100 participants (50 with and 50 without anemia) will be tested. Remaining samples will be stored for future analysis. Approximately 2.5cc of blood will be taken from infants for these additional studies.

Due to constraints on resources, the number of tests may need to curtailed. If funds (or blood volume) become a constraint, test will be prioritized as follows: folate, B12, iron, iron binding capacity, and vitamin A.

7.15 <u>Stored samples</u>
Remaining blood from study specimens will be stored for potential future use. Patients will be asked specifically during the consenting process if they would consent for their samples to be stored and studied in the future. Remaining samples will be destroyed at the end of this study, for participants who decline that these samples be stored for future HIV-approved research. Prior to using these samples for studies outside of the scope of this protocol, approval will be sought from the institutional review boards (IRB).

7.16 <u>Infant feeding</u>
Counseling regarding feeding will be provided in accordance with recommendations by the Botswana Government. Women will choose their feeding method, with supportive counseling. Formula feeding and weaning will be accompanied by extensive counseling

about the safe preparation of infant formula. Among women who choose to breastfeed and choose not to wean at 6 months, CTX will be continued beyond the 6-month visit and participants will be counseled how to obtain services from the Botswana Government health centers.

7.17 Participants who do not follow-up

Women who have consented to participate but fail to follow-up with the Study staff will be visited at their homes (if permission for this was given at the time of HIV screening) and encouraged to go to the study clinic for enrollment or follow-up.

7.18 Study Termination

The study may be terminated by the Botswana Government/Ministry of Health, by IRBs (HRDC and HSC), the sponsor (NIH DAIDS)or by the study team, if new information (from this study or other studies) emerges that affects the risk/benefit balance of this infant CTX prophylaxis study. Participants who stop the study early will be asked to participate in one final study visit at which final clinical data and FBC with differential will be collected.

7.19 Integration with Botswana National Program

Enrolled participants, both mothers and infants, will be actively encouraged and urged to concurrently obtain care from the Botswana National Program, and will be assisted with appropriate referrals. Education about services offered at the National Program, including vaccination, HAART, illness evaluation, will be provided. Urgent referrals to the National Program, made in person if applicable, will be made for infants testing positive for HIV or meeting WHO clinical criteria (stage 2, 3, or 4). Initiation of HAART is not a contraindication for continued participation in the study.

In order to improve communication, enrolled participants will be given a letter explaining the study to give to practitioners in the National Program. The letter will explain that after 6 months of age the standard Botswana criteria for CTX prophylaxis should be used. Laboratory results will also be provided.

7.20 Schedule of Evaluations

INFANTS	Birth	1 month	3 months***	6 months
CRF Questionnaire (maternal/infant clinical history)	X	X	X***	X
Growth indices, physical exam as needed	X	X	X***	X
Hematology*		X***	X***	X***
Anemia Studies**			X***	X***
HIV DNA PCR		X	X***	X***

* Hematology includes CBC with differential, i.e. hemoglobin, hematocrit, RBC, MCV, WBC, Platelets, automated differential.
** Anemia studies, in infants with grade 3 or 4 anemia and an equal number (selected randomly) of approximately 50 infants without anemia, may include: iron, iron binding capacity, vitamin B12, vitamin A, folate, and/or blood smear.
*** These visits / evaluations will only be conducted for infants in cohort A, and NOT for infants in cohort B.

The maximum amount of blood to be drawn at any visit would be less than 5 mL.

7.21 Accrual
Patients will be enrolled sequentially from mothers recruited from maternity wards, with goal of 2 subjects daily per site, until enrollment targets have been met. To facilitate parallel comparison with the breastfeeding cohort of Mma Bana whose mothers initiated HAART during pregnancy, the recruitment for cohort A will prioritize HIV- and HAART-exposed infants as follows:

1. Breastfeeding infants born to mothers who initiated HAART for first time during pregnancy, by 35 weeks gestation
2. Breastfeeding feeding infants born to mothers who re-initiated HAART during pregnancy, by 35 weeks gestation
3. Breastfeeding infants born to mothers who initiated or re-initiated HAART during pregnancy, but after 35 weeks gestation
4. Breastfeeding feeding infants born to mothers who were on HAART at time of conception and continued during pregnancy
5. Formula-feeding infants born to mothers who initiated HAART for first time during pregnancy
6. Formula-feeding infants born to mothers who re-initiated HAART during pregnancy
7. Formula-feeding infants born to mothers who initiated or re-initiated HAART during pregnancy, but after 35 weeks gestation
8. Formula-feeding infants born to mothers who were on HAART at time of conception and continued during pregnancy

The targeted enrollment is 222 infants in cohort A and another 222 infants in cohort B.

7.18 Off Study
Following are the reasons for discontinuation from study participation:
- Removal of consent
- Lost to follow-up
- Completion of study follow-up as defined by the protocol
- Premature closure of the study or of assigned treatment arms by the Botswana Health Research and Development Committee (HRDC), the Harvard School of

Public Health (HSPH) Human Subjects Committee (HSC) or Partners IRB, the Botswana Ministry of Health, the United States National Institutes of Health Division of AIDS, or the study team

- Refusal by study participant to continue any contact for follow-up
- Death of participant

Note: When a participant goes Off Study a final blood draw (for hematology) will be requested. After obtaining sample or refusal, all data collection ends.

8 Safety and Toxicity

For infants in cohort A, we will be monitoring infant hematologic toxicities and rash, specifically.

8.1 General management of grade 1-2 toxicities

Infants who develop a Grade 1-2 toxicity can be continued on CTX at the same dose, with closer follow-up / monitoring.

8.2 General management of grade 3-4 toxicities

Infants who develop a Grade 3-4 symptomatic toxicity will generally have to have the CTX interrupted/stopped. Infants who develop an asymptomatic Grade 3 laboratory toxicity only may have the CTX continued with close follow-up monitoring (please see details below). Infants with grade 3 or 4 adverse events will be followed until resolution even if beyond the planned 6 month study period or until condition determined to be chronic/permanent by study investigator(s).

8.3 Management of rash

For grade 1 (erythema/localized maculopapular) or grade 2 (diffuse maculopapular) rash, CTX can be continued with careful observation (generally at least weekly, until rash stabilizes or resolves). In the event of a Grade 3 (vesicles, bullae, limited superficial mucosal involvement) or Grade 4 (extensive bullae, Stevens Johnson syndrome, TEN, or extensive mucosal involvement) rash, CTX should be permanently discontinued (and not restarted).

8.4 Management of anemia

CTX can be continued without any change in infants with ≤ Grade 1-2 anemia. CTX can be continued with hemoglobin monitoring every 2-4 weeks in asymptomatic children with Grade 3 anemia. CTX should be immediately discontinued in children with symptomatic Grade 3 anemia, or any Grade 4 anemia. If the CTX is deemed to be the most likely cause of the symptomatic Grade 3 anemia or asymptomatic/symptomatic Grade 4 anemia, the CTX should be permanently discontinued. Children who develop a symptomatic Grade 3 or any Grade 4 anemia deemed to be related to causes other than CTX should have the CTX stopped and held until the anemia is ≤ Grade 2 (at which point the CTX can be reinitiated, with careful follow-up of hemoglobin).

8.5 Management of neutropenia

CTX can be continued without any change in infants with <= Grade 2 neutropenia. CTX can be continued, with monthly measurements of FBC/differential, in children with Grade 3 neutropenia. CTX should be permanently discontinued in children with Grade 4 neutropenia.

8.6 Toxicity management in cohort B
The study will not provide CTX to infants in cohort B. However, study staff will be available for medical consultative services for children in cohort B, and will provide urgent care and facilitate prompt referral for ill children.

8.7 Infants with positive HIV-1 DNA PCR
In general, results of the infant birth and 1-month HIV-1 DNA PCRs will be available within 1-2 weeks of submission. If they are positive, a second (confirmatory) PCR should be obtained as soon as feasible.

Infants who test positive for HIV-1 infection will be referred personally to the Botswana National Program for evaluation and consideration of treatment. Referral will be made immediately without waiting for confirmatory PCR. If agreeable to the mother, the infant will continue to participate in this CTX safety study. However, after confirmation no further HIV PCR measurements will be performed. Infant caregivers will be urged to continue infant CTX prophylaxis among HIV-infected infants. HIV-infected infants will continue to be followed on-study.

9 Study management

9.1 Study medication accountability
Botswana-Harvard Partnership (BHP) pharmacists/pharmacy technicians will be responsible for acquiring, storing, and maintaining records of supply of CTX. Medication will be dispensed by study medical officer or study nurse and documented. Dispensing records will be reconciled with pharmacy records by BHP pharmacist/pharmacy technician.

9.2 Reporting requirements

The BHP DMC will oversee the data entry and management.

The following will be reported on CRFs:

9.2.1: Mother: demographics/SES/food security, obstetric, delivery, HIV, ARV, concomitant medication, medical history, feeding counseling and choice (at enrollment only), attitudes toward health system, and death occurring on-study.

9.2.2 Child

Cohort A only: All grade 3 or 4 diagnoses or signs/symptoms at enrollment and occurring on-study (all diagnoses, signs and symptoms regardless of grade will be recorded on source documents); concomitant prescription and traditional medications, and multivitamin/iron/folate supplements

Cohort B: CTX is the only reportable concomitant medication

All HIV-infected infants, all HIV-related signs/symptoms/diagnoses, regardless of grade.

All hospitalizations (including prolongation of initial delivery-associated hospitalization, due to infant illness)

All deaths

Feeding method, information regarding who is primarily caring for the child,

All protocol-required laboratory results (including values of repeat hematology testing performed for purposes of toxicity resolution monitoring), regardless of grade; and all additional laboratory results at grade 3 or above.

9.3 Adverse Event reporting

The Standard Level of EAE reporting (see Department of Acquired Immunodeficiency Syndrome [DAIDS] Manual for Reporting of Adverse Events to DAIDS, dated May 6, 2004) will be used, for reporting to the DAIDS. Given that the Standard Level of reporting is used, the EAEs (summarized below) will be reported in real time to DAIDS regardless of "expectedness". EAEs will be reported to the DAIDS within 3 working days of the site becoming aware of the event. EAE/SAE reporting will only be performed for the child, who is the study participant in this study (EAEs/SAEs will not be reported on caregivers).

Definition of Expedited Adverse Events (EAEs) for reporting in real time to DAIDS:

- All deaths
- All congenital anomalies/birth defects/fetal losses (these reports will be very rare, as only liveborn infants without severe congenital anomaly are enrolled)
- All disabilities/incapacities
- All hospitalizations that are "suspected adverse drug reactions" (cannot rule out relationship to infant CTX)
- All other Grade 4 events that are "suspected adverse drug reactions" (SADRs) (cannot rule out relationship to infant CTX)
- All other adverse events that, based upon appropriate medical judgment, may jeopardize the subject's health and may require medical or surgical intervention to prevent one of the outcomes listed above

Reporting of EAEs/SAEs to IRBs will follow the requirements of each IRB. In general, *unexpected*, *study-related* EAEs are reported to the Harvard School of Public Health Human Subjects Committee (HSC) and the Botswana Human Research and Development Committee (HRDC) in real time (within 3 working days of the site becoming aware of the event). *Anticipated* SAEs (and SAEs that are deemed to be definitely *not* study-related) are generally reported to IRBs in the aggregate during annual re-approval applications. The Standard AE definition used above (which includes all traditionally-defined SAEs, as well as Grade 4 SADRs) will be used for reporting to IRBs as well.

AEs reported on an expedited basis will be documented on the DAIDS Expedited Adverse Event Reporting Form (EAE Reporting Form) available on the RCC website: http://rcc.tech-res-intl.com.

EAE Reporting Requirements for this Study

- EAE Reporting Level
  This study uses the Standard Level of expedited AE reporting as defined in the DAIDS EAE Manual.

- Study Agents for determining "relatedness" of EAEs:
  Study-provided cotrimoxazole (CTX) must be considered in determining relationships of AEs requiring expedited reporting to DAIDS (CTX provided outside of the study for cohort B is not considered study drug).

- Grading Severity of Events
  The Division of AIDS Table for Grading the Severity of Adult and Pediatric Adverse Events (DAIDS AE Grading Table), Version 1.0, December, 2004, will be used and is available on the RCC website at http://rcc.tech-res-intl.com/.

- EAE Reporting Periods
  AEs must be reported on an expedited basis at the Standard Level during the Protocol-defined EAE Reporting Period, The EAE Reporting Period is the entire study duration for an individual subject (from study enrollment until study completion or discontinuation of the subject from study participation for any reason). After the end of the Protocol-defined EAE Reporting Period, sites must report serious, unexpected, clinical suspected adverse drug reactions if the study site staff becomes aware of the event on a passive basis, i.e., from publicly available information.

9.4 Early termination of the study for adverse events, cohort A

Because this is an observational study of the rate of hematologic toxicities of an exceedingly commonly-used, approved antibiotic at standard prophylactic doses, a Safety Monitoring Committee has not been created. Rather, we will discuss with the IRBs and the DAIDS medical officer whether or not the study should be stopped early if the absolute rate of Grade 3 or 4 anemias/neutropenias on CTX exceeds 25% among either breastfeeding or formula feeding infants after at least 50 infants have been enrolled in that feeding group. As noted below, prophylactic CTX to breastfeeding infants born to HIV-infected women is standard of care in Botswana and throughout the world. CTX is often given for many months to formula-fed children born to HIV-infected women while definitive infant diagnostics are awaited, but is not standard of care when infant HIV infection can be ruled out by early HIV-1 DNA PCR (although studies of CTX prophylaxis among these HIV-uninfected infants, to prevent excess morbidity and mortality, are being planned in several sites). It is therefore this latter group of formula-fed infants for whom the determination of cessation of CTX prophylaxis as part of this study is most relevant.

9.5 Timeline

Recruitment and enrollment February 2009 to August 2009. Completion of follow-up February 2010. Data compilation and analysis to be complete by March 2010 and preparation for presentation in July, 2009.

10 Human subjects

We will adhere to the ethical principles and guidelines for the protection of human subjects of research as detailed in the Belmont Report. Each mother will have of her own free will given written informed consent prior to study participation. The informed consent process/forms will include detailed descriptions of the risks and benefits to the infant, will be translated into Setswana (the national language of Botswana) and then back translated into English by someone unfamiliar with the original form and compared for accuracy.

Mothers and infants in both cohorts will receive standard treatment as recommended by the WHO and the Ministry of Health of Botswana. The only exception to this is the provision of extended infant CTX in *formula-fed*, HIV-uninfected infants in cohort A (which occurs functionally for many children in Botswana due to delayed diagnostics, but

which would not be necessary in the context of early HIV-1 DNA PCR). There is a hope that CTX prophylaxis of formula-fed infants born to HIV-infected women will help prevent some infant morbidity and mortality related to regular bacterial/parasitic pathogens, but this has not been proven (the hope is that this study will provide safety data to help inform a study of the efficacy of infant CTX prophylaxis at the time of formula feeding/weaning among HIV-uninfected infants born to HIV-infected women). The use/dosing of CTX in this age range/population is not experimental. With regard to beneficence specifically as it relates to children, it is our opinion that the benefits of the study outweigh the risks of phlebotomy (which is safe and minimally uncomfortable in trained hands),CTX exposure, and inadvertent disclosure of health information (expected to be rare given incorporated protections).

Participants will be compensated for work time and travel costs at standard rates, as endorsed by the Botswana Ethics Committee (HRDC).

Approvals for human subject research will be sought from the Health Research Development Committee (Ethics Committee) at the Botswana Ministry of Health, and the institutional review boards of the Harvard School of Public Health, Partners Healthcare, (reciprocity agreements may apply, between Harvard School of Public Health and Partners IRBs).

Participants will be women (mothers) and their children (both female and male).

11 Analysis and statistical considerations

11.1 Endpoints

The primary endpoint of cohort A is the proportion of infants experiencing grade 3 (severe) or 4 (life-threatening) anemia occurring between 1 and 6 months of age in infants who were exposed to *in utero* HAART (i.e., grade 3 or 4 anemia occurring after study entry, and excluding the entry pre-CTX values). We will use the "DAIDS Table for Grading the Severity of Adult and Pediatric Adverse Events" from 2004, and will consider the occurrence of a grade 3 or 4 anemia on any one occasion during follow-up to be a study endpoint.

For the evaluation of risk factors for early morbidity/mortality in HIV-exposed infants (combined cohorts A and B), death and a combined end-point of death and/or hospitalization will be used.

Secondary endpoints include the incidence of grade 3 or 4 neutropenia and the composite endpoint of grade 3 or 4 infant diagnoses/hospitalizations/death occurring between 1 and 6 months of age.

11.2 Sample size, hematologic safety of CTX (cohort A)

We are interested in comparing the proportion experiencing grade 3 / 4 anemia between infants who receive CTX and infants who did not receive CTX (in infants born to HIV-infected women with baseline CD4 < 250 cells/mm^3 in the Mma Bana cohort). Based upon existing preliminary data from the Mma Bana cohort, approximately 6-10% of infants have experienced at least one episode of grade 3 /4 anemia between 1 and 6 months of life. We wish to detect an absolute 10 percentage points or greater increase in the incidence of grade 3 / 4 anemia, e.g. from 8% to 18%. Secondary endpoints include grade 3 / 4 neutropenia (1-6 months), prevalence of nutrient deficiencies in anemic infants, and severe morbidity/mortality through 6 months.

The sample size calculation is based on the primary endpoint. In order to detect an absolute 10 percentage point increase (from 8 to 18%) in the incidence of severe or life-threatening anemia with 80% power using a two-sided Fisher's exact test with a Type I error rate of 0.05, we will require complete data from approximately 200 infants receiving CTX in the new cohort, and 170 infants from the existing Mma Bana observational cohort. However, the incidence of anemia is not yet known in either group. The table below provides sample size for various underlying rates of anemia among breastfeeding infants (using 2-sided Fisher's exact test, alpha 0.05):

**Percentage with grade 3 or4 anemia between 1 to 6 mos**			
**Mma Bana (no CTX)**	**New cohort (CTX)**	**CTX infants, cohort A**	**Mma Bana infants**
4	14	114	170
6	16	146	170
**8**	**18**	**200**	**170**
10	20	250	170

To allow for a 10% loss to follow-up and incomplete data, we plan to accrue 222 infants in cohort A.

11.3 <u>Sample Size, risk factors for early morbidity/mortality (cohorts A and B)</u>
In the Mashi study, 8% of infants in the formula arm and 3.4% of infants in the breastfeeding arm had died by 6 months postpartum; and ~13% of infants in the formula arm (and 9% of infants in the breastfeeding arm) had been hospitalized by 6 months of age. It is important to note that currently, the vast majority of HIV-infected women are formula-feeding their infants For purposes of sample size estimates, we assume (somewhat conservatively) that overall, approximately 15% of infants in this predominantly formula-feeding study will either die or be hospitalized by 6 months of age.

The sample size is calculated to have a reasonable opportunity (80 percent power) to detect factors (p= 0.05) associated with a clinically significant increase in mortality (Hazard Ratios to 2.0 to 3.0).   With evaluable data from 400 infants enrolled in both cohorts A and B, the proportion of the population with a factor associated with increased risk could account for as little as one-fifth of the study population (if the predictor is present in >20% of participants, then power increases). The analysis would not have sufficient power to reliably identify risks present in smaller proportions or for hazard ratios less than 2.1. However, logistical challenges of recruiting additional patients are not outweighed the small marginal improvement in power. Please see table below:

Proportion of total population with hospitalization and/or death	Proportion of population with risk factor	Hazard Ratio	Evaluable sample size	Power
0.15	0.2	3.0	400	0.99
0.15	0.2	2.67	400	0.98
**0.15**	**0.2**	**2.1**	**400**	**0.81**
0.15	0.2	2.0	400	0.74
0.15	0.2	2.0	465	0.80
0.2	0.2	2.0	400	1.0
0.1	0.2	2.0	400	0.55
0.1	0.2	2.0	465	0.61
0.2	0.1	2.0	465	0.57

With an estimated 10 percent lost to follow-up and incomplete data, we aim to enroll a total of 444 infants in order to have data on 400 participants. We aim to enroll 222 infants into cohort A, and these infants will contribute data to this analysis. An additional 222 infants will be recruited, into cohort B, for the purposes of this analysis.

The vast majority (nearly all) infants are expected to be formula-fed for the foreseeable future, barring major programmatic changes.

11.4 Data inclusion

Events (neutropenia and anemia) at enrollment, month 1 (prior to CTX administration), will not be considered in the primary analysis. Infants who acquire HIV and any child who starts CTX as part of the study (even if he/she must stop it due to toxicity) will be included in the primary, intent-to-treat analysis.

Data from infants of historical cohorts (Mma Bana and Mashi) will be used for comparision with the newly enrolled cohorts. Data from historical cohorts will not be included for analysis if infants do not meet entrance criteria for the current study. All data from infants meeting shared entry criteria (both maternal/infant), will be included.

11.5 Methods of analysis

The primary and secondary endpoints (proportion of infants experiencing endpoint between 1 and 6 months) will be compared between the two cohorts using Fisher’s exact test.

The continuous change in hemoglobin from enrollment to 3 and 6 months will also be compared between CTX exposed and non-exposed infants using Wilcoxon rank-sum tests. The test is a non-parametric alternative to the two-sample t-test and should be used whenever the assumptions that underlie the *t*-test cannot be satisfied**.** Multivariate regression analyses will be used to assess whether clinical and demographic factors affect the comparison of the study endpoints. The following factors will be among those considered in our multivariate regressions analysis: infant HIV infection status, sex, gestational age at birth, infant weight, duration of maternal HAART, and feeding strategy.

Evaluation of the effect of CTX on the composite endpoint of infant grade 3 or 4 morbidity, hospitalization, or mortality will be performed using Fisher’s exact test.

The proportions of formula-fed infants experiencing grade 3 or 4 anemia and neutropenia between 1 and 6 months on CTX in this study will be described (with 95% CIs). Rates of hematologic toxicity will be compared with rates among formula-fed infants born in the Mashi study (and not receiving CTX prophylaxis), although the Mashi participants were largely not exposed to maternal HAART *in utero.*

The evaluation of risk factors for early morbidity/mortality will utilize time to event outcomes modeled using Cox regression. We will explore significant factors influencing the primary endpoints via a univariate model and then fit a multivariate model. Covariates may include maternal disease status and SES factors, infant HIV infection status, feeding modality, infant CTX receipt, locale, among others.

11.6 <u>Death and loss-to-follow-up</u>

Loss to follow-up (LFU) is expected to be minimal. In prior studies at these sites, less than 2% of infants between 1-6 months age were LFU. We are using the same procedures and anticipate approximately the same rate. Mortality rates are unfortunately 4% of breast-fed and 8% of formula-fed children during this period. Whether CTX will lower mortality in non-HIV infected infants is unknown. We estimate that 6-10% of infants will die or be LFU during the study period.

Last known, non-baseline, data will be used in analysis of primary and secondary endpoints. Infants with only baseline data will be excluded from analysis. Sensitivity analyses will be performed to evaluate for possibility of death or LFU lead to bias in findings. A second composite endpoint (new grade 3 or 4 anemia, grade 4 neutropenia, death, or loss to follow-up) will also be compared between groups, to incorporate toxicity and morbidity/mortality data as well as LFU.

11.7 <u>Bias and confounding</u>

In designing this study, every attempt has been made to make the new cohort as similar to Mma Bana as possible, so as to limit possibility for bias. The newly enrolled cohort will be drawn from the same clinics and meet similar inclusion/exclusion criteria as were the comparison cohorts for Mma Bana and Mashi. The follow-up periods for infants will overlap temporally. The standard / type of care provided to children in this age range is has not changed significantly. The same case report forms will be used and many of the same staff members will be employed for the new cohort. The cohorts are therefore expected to be quite similar and bias related to these factors should be minimized.

Despite prioritization of enrollment of eligible breast-feeding infants, the cohort A is likely to be a predominantly formula-fed group of infants given the increasing (over time) preference for HIV-exposed infants to be formula-fed in Botswana. Comparison of this cohort A with the breast-feeding Mma Bana cohort may be confounded by a possible effect of feeding method on the incidence of severe anemia. In resource-rich settings, formula feeding has been found to be protective against iron deficiency and anemia,[51] and it is common practice to provide breast-feeding infants with nutrient supplementation. In neighboring Zambia, iron deficiency was found to be common at birth and was not prevented by supplementary foods, frequently leading to anemia by six-months of age.[52] It is anticipated that formula feeding would prevent iron deficiency and consequently anemia. This effect may make the detection of a possible role of CTX potentiating anemia when compared with the Mma Bana cohort, which has not received routine iron supplementation, more difficult. It is also possible, though not likely, that formula-feeding could bias results in the opposite direction. Increased rates of anemia due to more frequent acute illnesses (e.g. diarrhea, pneumonia) or due to allergic

enterocolitis (e.g. cow's milk allergy) could occur and could be falsely attributed to CTX (malaria will not be an issue, given minimal malaria in the study region).

As most infants will be either be CTX- and formula-exposed or no-CTX and breast-milk exposed, it will not be possible to control the effect of feeding method on anemia through multivariate analysis and this is a limitation of the study design. However, if a substantial number of breast-feeding infants can be enrolled in CTX cohort, it may be possible to estimate this effect. Furthermore, diagnoses that could contribute to anemia— bleeding, recurrent infection, and enterocolitis— will be recorded and can be analyzed. In the randomized Mashi trial no significant differences in incidence of severe anemia was observed between breast-feeding and formula-feeding cohorts.

Additional measured and unmeasured differences may exist between the cohorts. Baseline characteristics will be compared to evaluate for unexpected differences. Data will be collected permitting creation of a propensity score model. This model could be used to perform sensitivity analyses to evaluate whether the comparison cohorts have similar characteristics if concerns about differences arise.

Cohorts A and B will be combined for analysis of thesecond primary aim— evaluating predictors of early morbidity and mortality among formula-feeding infants. Cohorts A and B will differ in some important characteristics. Infants in cohort A will all be exposed to HAART and will be followed more closely in a study clinic, whereas only some infants in cohort B will be exposed to HAART and will receive the bulk of their care from government clinics. In addition, infants in cohort A will all receive 6 months of CTX and infants in cohort B will receive CTX only until HIV-infection can be excluded (2-6 months). These differences will enable construction of a multivariate model to evaluate independent predictors of early morbidity and mortality.

12 Vertebrate animals

Not applicable.

13 Literature cited

1. *Towards universal access: scaling up priority HIV/AIDS interventions in the health sector: progress report, April 2007.* Geneva: World Health Organization, UNAIDS, UNICEF;2007.

2. *Guidelines on co-trimoxazole prophylaxis for HIV-related infections among children, adolescents, and adults: recommendations for a public health approach.* Geneva: World Health Organization;2006.

3. Feiterna-Sperling C, Weizsaecker K, Buhrer C, et al. Hematologic effects of maternal antiretroviral therapy and transmission prophylaxis in HIV-1-exposed uninfected newborn infants. *J Acquir Immune Defic Syndr.* May 1 2007;45(1):43-51.

4. Le Chenadec J, Mayaux MJ, Guihenneuc-Jouyaux C, Blanche S. Perinatal antiretroviral treatment and hematopoiesis in HIV-uninfected infants. *AIDS.* Sep 26 2003;17(14):2053-2061.

5. Pacheco SE, McIntosh K, Lu M, et al. Effect of perinatal antiretroviral drug exposure on hematologic values in HIV-uninfected children: An analysis of the women and infants transmission study. *J Infect Dis.* Oct 15 2006;194(8):1089-1097.

6. *Antiretroviral drugs for treating pregnant women and preventing HIV infection in infants: towards universal access: recommendations for a public health approach.* Geneva: World Health Organization;2006.

7. Tapp H, Savarirayan R. Megaloblastic anaemia and pancytopenia secondary to prophylactic cotrimoxazole therapy. *J Paediatr Child Health.* Apr 1997;33(2):166-167.

8. Moyle G, Sawyer W, Law M, Amin J, Hill A. Changes in hematologic parameters and efficacy of thymidine analogue-based, highly active antiretroviral therapy: a meta-analysis of six prospective, randomized, comparative studies. *Clin Ther.* Jan 2004;26(1):92-97.

9. Kline MW, Van Dyke RB, Lindsey JC, et al. A randomized comparative trial of stavudine (d4T) versus zidovudine (ZDV, AZT) in children with human immunodeficiency virus infection. AIDS Clinical Trials Group 240 Team. *Pediatrics.* Feb 1998;101(2):214-220.

10. Ssali F, Stohr W, Munderi P, et al. Prevalence, incidence and predictors of severe anaemia with zidovudine-containing regimens in African adults with HIV infection within the DART trial. *Antivir Ther.* 2006;11(6):741-749.

11. WHO/UNICEF/UNU. *Iron deficiency anaemia: assessment, prevention, and control.* Geneva: World Health Organization;2001.

12. *Guidelines for the use of iron supplements to prevent and treat iron deficiency anemia.* Geneva and Washington, DC: WHO and UNICEF;1998.

13. Stoltzfus RJ. Iron-deficiency anemia: reexamining the nature and magnitude of the public health problem. Summary: implications for research and programs. *J Nutr.* Feb 2001;131(2S-2):697S-700S; discussion 700S-701S.

14. English M, Ahmed M, Ngando C, Berkley J, Ross A. Blood transfusion for severe anaemia in children in a Kenyan hospital. *Lancet.* Feb 9 2002;359(9305):494-495.

15. Berkley JA, Ross A, Mwangi I, et al. Prognostic indicators of early and late death in children admitted to district hospital in Kenya: cohort study. *BMJ.* Feb 15 2003;326(7385):361.

16. Brabin BJ, Premji Z, Verhoeff F. An analysis of anemia and child mortality. *J Nutr.* Feb 2001;131(2S-2):636S-645S; discussion 646S-648S.

17. Brabin B, Prinsen-Geerligs P, Verhoeff F, Kazembe P. Anaemia prevention for reduction of mortality in mothers and children. *Trans R Soc Trop Med Hyg.* Jan-Feb 2003;97(1):36-38.

18. Calis JC, Phiri KS, Faragher EB, et al. Severe anemia in Malawian children. *N Engl J Med.* Feb 28 2008;358(9):888-899.

19. *National guidelines for the prevention of mother-to-child transmission of HIV, in press*: Ministry of Health, Botswana;2007.

20. Mother-to-child transmission of HIV infection in the era of highly active antiretroviral therapy. *Clin Infect Dis.* Feb 1 2005;40(3):458-465.

21. Thior I, Lockman S, Smeaton LM, et al. Breastfeeding plus infant zidovudine prophylaxis for 6 months vs formula feeding plus infant zidovudine for 1 month to reduce mother-to-child HIV transmission in Botswana: a randomized trial: the Mashi Study. *JAMA.* Aug 16 2006;296(7):794-805.

22. Shapiro RL, Holland DT, Capparelli E, et al. Antiretroviral concentrations in breast-feeding infants of women in Botswana receiving antiretroviral treatment. *J Infect Dis.* Sep 1 2005;192(5):720-727.

23. Graham SM. Prophylaxis against Pneumocystis carinii pneumonia for HIV-exposed infants in Africa. *Lancet.* Dec 14 2002;360(9349):1966-1968.

24. Walker AS, Mulenga V, Ford D, et al. The impact of daily cotrimoxazole prophylaxis and antiretroviral therapy on mortality and hospital admissions in HIV-infected Zambian children. *Clin Infect Dis.* May 15 2007;44(10):1361-1367.

25. Kafulafula G, Thigpen M, Hoover D, et al. Post-weaning Gastroenteritis and Mortality in HIV-uninfected African Infants Receiving Antiretroviral Prophylaxis to Prevent MTCT of HIV-1. Paper presented at: Conference on Retroviruses and Opportunistic Infections (CROI)2007; Los Angeles.

26. Thomas T, Masaba R, van Eijk A, et al. Rates of Diarrhea Associated with Early Weaning among Infants in Kisumu, Kenya. Paper presented at: Conference on Retroviruses and Opportunistic Infections (CROI)2007; Los Angeles.

27. Athena Kourtis DF, L Hyde, H C Tien, C Chavula, N Mumba, M Magawa, R Knight, C Chasela, C van der Horst, and the BAN Study Team. Diarrhea in Uninfected Infants of HIV-infected Mothers Who Stop Breastfeeding at 6 Months: The BAN Study Experience. Paper presented at: Conference on Retroviruses and Opportunistic Infections (CROI)2007; Los Angeles.

28. Mermin J, Lule J, Ekwaru JP, et al. Cotrimoxazole prophylaxis by HIV-infected persons in Uganda reduces morbidity and mortality among HIV-uninfected family members. *AIDS.* Jul 1 2005;19(10):1035-1042.

29. Imrie KR, Prince HM, Couture F, Brandwein JM, Keating A. Effect of antimicrobial prophylaxis on hematopoietic recovery following autologous bone marrow transplantation: ciprofloxacin versus co-trimoxazole. *Bone Marrow Transplant.* Feb 1995;15(2):267-270.

30. Woods WG, Daigle AE, Hutchinson RJ, Robison LL. Myelosuppression associated with co-trimoxazole as a prophylactic antibiotic in the maintenance phase of childhood acute lymphocytic leukemia. *J Pediatr.* Oct 1984;105(4):639-644.

31. Moh R, Danel C, Sorho S, et al. Haematological changes in adults receiving a zidovudine-containing HAART regimen in combination with cotrimoxazole in Cote d'Ivoire. *Antivir Ther.* 2005;10(5):615-624.

32. Toure S, Gabillard D, Inwoley A, Seyler C, Gourvellec G, Anglaret X. Incidence of neutropenia in HIV-infected African adults receiving co-trimoxazole prophylaxis: a 6-year cohort study in Abidjan, Cote d'Ivoire. *Trans R Soc Trop Med Hyg.* Aug 2006;100(8):785-790.

33. Shah MM, Li Y, Christensen RD. Effects of perinatal zidovudine on hematopoiesis: a comparison of effects on progenitors from human fetuses versus mothers. *Aids.* Sep 1996;10(11):1239-1247.

34. Cooper ER, Charurat M, Mofenson L, et al. Combination antiretroviral strategies for the treatment of pregnant HIV-1-infected women and prevention of perinatal HIV-1 transmission. *J Acquir Immune Defic Syndr.* Apr 15 2002;29(5):484-494.

35. Siepone K. Towards Elimination of Paediatric AIDS. Paper presented at: 2nd Botswana International HIV Conference2008; Gaborone, Botswana.

36. Newell ML. Current issues in the prevention of mother-to-child transmission of HIV-1 infection. *Trans R Soc Trop Med Hyg.* Jan 2006;100(1):1-5.

37. Coovadia HM, Rollins NC, Bland RM, et al. Mother-to-child transmission of HIV-1 infection during exclusive breastfeeding in the first 6 months of life: an intervention cohort study. *Lancet.* Mar 31 2007;369(9567):1107-1116.

38. Mbori-Ngacha D, Nduati R, John G, et al. Morbidity and mortality in breastfed and formula-fed infants of HIV-1-infected women: A randomized clinical trial. *Jama.* Nov 21 2001;286(19):2413-2420.

39. Becquet R, Bequet L, Ekouevi DK, et al. Two-year morbidity-mortality and alternatives to prolonged breast-feeding among children born to HIV-infected mothers in Cote d'Ivoire. *PLoS Med.* Jan 2007;4(1):e17.

40. WHO/UNICEF. New Data on the Prevention of Mother-to-Child Transmission of HIV and their Policy Implications: World Health Organization; 2000.

41. Botswana 2008 National HIV/AIDS Guidelines: Ministry of Health, Botswana; 2008.

42. Jackson DJ, Chopra M, Doherty TM, et al. Operational effectiveness and 36 week HIV-free survival in the South African programme to prevent mother-to-child transmission of HIV-1. *Aids.* Feb 19 2007;21(4):509-516.

43. Doherty T, Chopra M, Jackson D, Goga A, Colvin M, Persson LA. Effectiveness of the WHO/UNICEF guidelines on infant feeding for HIV-positive women: results from a prospective cohort study in South Africa. *Aids.* Aug 20 2007;21(13):1791-1797.

44. Chopra M, Rollins N. Infant feeding in the time of HIV: rapid assessment of infant feeding policy and programmes in four African countries scaling up prevention of mother to child transmission programmes. *Arch Dis Child.* Apr 2008;93(4):288-291.

45. Effect of breastfeeding on infant and child mortality due to infectious diseases in less developed countries: a pooled analysis. WHO Collaborative Study Team on the Role of Breastfeeding on the Prevention of Infant Mortality. *Lancet.* Feb 5 2000;355(9202):451-455.

46. Brahmbhatt H, Kigozi G, Wabwire-Mangen F, et al. Mortality in HIV-infected and uninfected children of HIV-infected and uninfected mothers in rural Uganda. *J Acquir Immune Defic Syndr.* Apr 1 2006;41(4):504-508.

47. Kilewo C, Karlsson K, Ngarina M, et al. MITRA Plus: Prevention of mother-to-child transmission for HIV-1 through breastfeeding by treating mothers prophylactically with triple antiretroviral therapy in Dar es Salaam, Tanzania. Paper presented at: 4th IAS Conference on HIV Pathogenesis, Treatment, and Prevention2007; Sydney, Australia.

48. Arendt V, Ndimubanzi P, Vyankandonera J, et al. AMATA study: effectiveness of antiretroviral therapy in breastfeeding mothers to prevent post-natal vertical transmissionin Rwanda. Paper presented at: IAS2007; Sydney, Australia.

49. Lockman S, Shapiro RL, Smeaton LM, et al. Response to antiretroviral therapy after a single, peripartum dose of nevirapine. *N Engl J Med.* Jan 11 2007;356(2):135-147.

50. Lockman S, Smeaton LM, Shapiro R, et al. Risk Factors for and Timing of Infant Mortality Among Children in a Randomized Infant Feeding Trial. Paper presented at: 15th Conference on Retroviruses and Opportunistic Infection2008; Boston, USA.

51. Chantry CJ, Howard CR, Auinger P. Full breastfeeding duration and risk for iron deficiency in U.S. infants. *Breastfeed Med.* Jun 2007;2(2):63-73.

52. van Rheenen PF, de Moor LT, Eschbach S, Brabin BJ. A cohort study of haemoglobin and zinc protoporphyrin levels in term Zambian infants: effects of iron stores at birth, complementary food and placental malaria. *Eur J Clin Nutr.* Dec 2008;62(12):1379-1387.
